# A Novel Prescription Digital Therapeutic Option for the Treatment of Metabolic Dysfunction-Associated Steatotic Liver Disease

**DOI:** 10.1016/j.gastha.2023.08.019

**Published:** 2023-10-01

**Authors:** Naim Alkhouri, Katherine Edwards, Mark Berman, Heather Finn, Rafael Escandon, Paul Lupinacci, Nicole Guthrie, Angie Coste, Jesus Topete, Mazen Noureddin

**Affiliations:** 1 Fatty Liver Program, Arizona Liver Health, Chandler, Arizona; 2 Better Therapeutics, San Francisco, California; 3 DGBI Consulting, LLC, Bainbridge Island, Washington; 4 Villanova University, Villanova, Pennsylvania; 5 Houston Methodist Hospital, Houston Research Institute, Houston, Texas

**Keywords:** Metabolic Dysfunction-Associated Steatotic Liver Disease, Metabolic Dysfunction-Associated Steatohepatitis, Prescription Digital Therapeutic, Cognitive Behavioral Therapy, Lifestyle Modification

## Abstract

**Background and Aims:**

Metabolic dysfunction-associated steatotic liver disease and metabolic dysfunction-associated steatohepatitis are pressing public health problems occurring alongside the rising prevalence of obesity and diabetes. This feasibility study explored the use of a novel prescription digital therapeutic (PDT) in this patient population.

**Methods:**

A prospective, open-label study was conducted at two hepatology clinics. Eligible patients had a baseline FibroScan controlled attenuation parameter >274 dB/m. Participants were given access to a PDT containing a novel form of cognitive behavioral therapy designed to treat cardiometabolic disease. Laboratory assessments, FibroScan, and magnetic resonance imaging proton density fat fraction (MRI-PDFF) imaging were conducted preintervention and postintervention.

**Results:**

Twenty-two participants were enrolled. Mean baseline fat fraction on MRI-PDFF was 18.7%. After the 90-day intervention, the mean relative reduction in MRI-PDFF was −16.2% (*P* = .011) in those with baseline PDFF ≥10%. Mean alanine transaminase decreased by −17.1 IU/L (*P* = .002). Participants achieved an average total body weight loss of −2.9% (*P* = .008) and controlled attenuation parameter score was reduced by −18.8 dB/m (*P* = .021). No serious or device-related adverse events were reported. An average improvement in health-related quality of life of +2.2 Healthy Days per month (*P* = .500) and high treatment satisfaction (mean Net Promoter Score of +75) were reported.

**Conclusion:**

After 90 days of digitally delivered cognitive behavioral therapy, improvements were observed in multiple endpoints without any adverse device effects. The safety, efficacy, and usability data observed strengthen the hypothesis that PDTs provide a scalable tool to address unmet behavioral treatment needs in metabolic dysfunction-associated steatotic liver disease and metabolic dysfunction-associated steatohepatitis (ClinicalTrials.gov number, NCT05357248).

## Background and Aims

Metabolic dysfunction-associated steatotic liver disease (MASLD) is a growing threat to public health and is considered the hepatic manifestation of metabolic syndrome.^1^^,^^2^ The condition currently affects an estimated 25%–30% of US adults,^3^^,^^4^ including approximately 70% of those with type 2 diabetes.^5^^,^^6^ The more advanced form of this condition, called metabolic dysfunction-associated steatohepatitis (MASH), affects approximately 5%–11% of American adults and has recently become a leading indication for liver transplant.^3^^,^^7^^,^^8^ The prevalence and sequelae of MASLD and MASH are projected to continue to grow,^9^ contributing to an increasing risk for patients and burden on the health-care system.

Despite the magnitude and morbidity of these conditions, there are currently no approved treatments. The standard of care guidelines for the prevention and management of MASLD and MASH emphasize the importance of facilitating lifestyle behavioral changes and reducing the cardiometabolic risk. Behavioral modifications resulting in weight loss, improved dietary quality, and increased physical activity have shown favorable effects on slowing, and even reversing, the progression of liver steatosis and fibrosis.^10^, ^11^, ^12^ Evidence supports targeted dietary interventions that replace the intake of highly processed foods, sugar-sweetened beverages, refined carbohydrates, red and processed meats, and other foods high in saturated fatty acids with a dietary pattern of whole, unprocessed plant-based foods rich in fiber and unsaturated fats with limited quantities of refined carbohydrates, red and processed meats.^13^, ^14^, ^15^

Despite this evidence, behavior change is notoriously difficult to facilitate and maintain in clinical practice. Providers struggle to provide effective behavioral counseling due to the lack of time and resources needed to help patients implement and maintain targeted, personalized behavioral therapy to improve MASLD or MASH.^16^ Moreover, many patients struggle to translate clinical recommendations into meaningful and sustainable behavioral changes in the absence of structure and support in their day-to-day lives.^17^

The current study evaluated a novel prescription digital therapeutic (PDT) for the treatment of cardiometabolic diseases using cognitive behavioral therapy (CBT). PDTs are a unique and emerging treatment category that delivers evidence-based therapeutic interventions to treat or manage a disorder using sophisticated software programs. These software programs are distinct from direct-to-consumer wellness apps as they are evaluated and authorized by the Food and Drug Administration, similar to a pharmaceutical agent of physical medical device, in order to ensure safety and effectiveness and require a prescription from a health-care provider in order to gain access. More detailed description of the nature of the PDT is provided later in the manuscript.

Use of this PDT has been associated with clinically meaningful outcomes in initial cohort trials in hypertension^18^ and type 2 diabetes.^19^^,^^20^ A multisite pivotal trial of the PDT significantly reduced hemoglobin A1c in participants randomized to the device compared to those receiving standard of care.^21^ Data from this trial led to authorization from the United States Food and Drug Administration as a PDT treatment indicated to provide an effective and safe form of CBT to patients 18 years or older with type 2 diabetes.

Both type 2 diabetes and hypertension have similar underlying metabolic etiologies to MASLD, including obesity, insulin resistance, and chronic meta-inflammation, which are directly related to healthy lifestyle behaviors.^13^^,^^22^ Accordingly, the objective of this study was to explore the potential for this novel digital platform to provide a safe, effective, and scalable treatment option for patients with MASLD and MASH. The secondary objective was to gather usability data and qualitative feedback from participants to inform tolerability, product refinement, and subsequent study planning.

## Methods

A prospective, open-label, proof-of-concept study, NCT05357248, was conducted at 2 specialty hepatology clinics that are part of the Arizona Liver Health research center. The study protocol was reviewed and approved by the institutional review board (Advarra, Columbia, MD) and all participants provided written informed consent.

Participants were recruited through the clinics’ existing patient population. Interested participants were screened for eligibility by medical history, baseline labs, biometrics, and focused physical exam. The study included 18–75-year-olds with a diagnosis of MASLD or MASH, confirmed by screening FibroScan (Echosens, Paris, France) controlled attenuation parameter (CAP) score >274 dB/m, with body mass index ≥30 kg/m^2^, and possession of smartphone (iPhone or Android) capable of running the PDT. Exclusion criteria included an inability to read or comprehend English (the PDT is only currently available in English), recent history of alcohol or substance abuse, or a history of other liver diseases. Those who met initial eligibility requirements received a baseline magnetic resonance imaging proton density fat fraction (MRI-PDFF) to further assess liver fat accumulation.

Eligible participants completed a call where they had the opportunity to ask questions before beginning the intervention. Participants were then sent a link to create an account and download the PDT from their smartphone app store. Treatment started after completion of a brief automated onboarding after which participants used the PDT at will. After 90 days, participants returned to the clinic to repeat the physical exam, biometrics, labs, FibroScan, and repeated the MRI-PDFF.

The primary outcome was the mean change in percent liver fat from baseline to 90 days for the subset of participants with an elevated baseline MRI-PDFF reading, defined as ≥10%.

The mean change from baseline in percent liver fat at day 90 was assessed in all enrolled subjects as a secondary endpoint. Other indicators of liver health, including alanine transaminase (ALT), CAP score, Fast™ score, FIB-4 index, and weight change, were also explored in all participants, regardless of baseline PDFF measurement. Change in MASH risk from baseline to end of intervention was assessed using change in Fast™ score categories (>0.67 = high risk for MASH, 0.67–0.35 = indeterminate, <0.35 = MASH is unlikely).

Bloodwork was collected at the respective clinic site and processed at one location of Quest Laboratories (Sonora, AZ). The CAP score and Fast™ score were measured using the FibroScan device (EchoSens, Paris, France) and performed by trained clinic staff. MRI-PDFF was performed by a regional imaging center (SMIL Southwest Medical Imaging) and all scans were overread by the same radiologist.

Treatment-emergent adverse events were solicited by the study staff, then evaluated and reported by the investigator. All data were collected via an electronic case report form (CRIO, Cambridge, MA).

Engagement metrics and study surveys were used to evaluate each participant’s degree of engagement with the PDT and usability of the platform. Health-related quality of life information was collected in the PDT at baseline and 90 days via the Centers for Disease Control and Prevention’s (CDC) Healthy Days Core Module (HRQOL-4) survey.^23^ Qualitative feedback on user experience was collected in the PDT with a Treatment Experience Survey and calls with the study sponsor’s support team were used to learn more about participant’s experience. The Net Promoter Score (NPS) was also dispensed in the PDT. This one-question survey asks users to rate on a scale of 1–10 how likely they are to recommend the treatment to a friend or loved one and generates a user satisfaction score between −100 and +100.^24^

### Investigational Prescription Digital Therapeutic

The investigational study device is a PDT delivered via a mobile application “app.” The device was created by the study sponsor, Better Therapeutics, Inc. The device delivers a novel form of CBT that targets individuals’ beliefs and behaviors related to improving health behaviors, such as diet and physical activity.

This therapeutic approach is unique from many other lifestyle change programs, such as diet or exercise plans, by taking a psychotherapeutic approach to drive cognitive shifts that lead to new patterns of thinking and living. The mechanism of action of CBT has been evidenced to be effective at changing behavior and improving glycemic control and other metabolic risk factors in many randomized controlled trials^25^, ^26^, ^27^ and the cortical changes elicited have been demonstrated to lead to lasting shifts in behavior and identify.

CBT results in behavioral change by:

1. Identifying maladaptive thoughts: creating awareness of core beliefs that could be holding people back from their personal goals and asserting that beliefs can change.
2. Introducing adaptive thoughts: stimulating introspection around maladaptive beliefs, creating alternative beliefs, and demonstrating how adopting them can bring about positive change.
3. Providing activities that bridge new ideas to new behaviors: integrating methods of behavioral activation so people can continuously practice behavior change and shift their beliefs in response to lived experiences.

The PDT is designed to help participants understand the steps they should prioritize by presenting a treatment plan that summarizes daily and weekly goals. All participants are exposed to the same set of lessons. Each lesson takes between 10 and 20 minutes to complete and skill exercises embedded in the lessons provide an opportunity for participants to practice new behavioral beliefs. A weekly goal-setting exercise uses an algorithm to help participants advance dietary and exercise change at a gradual and individualized pace.

Each week, the PDT asks participants to complete, a behavioral lesson and one or more skill-based exercises related to that week’s lesson. In addition to completing the core CBT activities each week, the PDT directs participants to self-report diet and exercise patterns, medication adherence, and self-monitored biometrics according to the patient’s comorbidities (eg, weight, blood pressure, and glucose). The treatment is delivered to participants without requiring additional participation from health-care providers.

The CBT platform evaluated was designed to focus broadly on beliefs and behaviors underlying many cardiometabolic diseases but was not specifically optimized for individuals with MASLD or MASH. This was intentional, as a secondary goal of the study was to learn from participants about therapeutic needs in this population and to inform the development of MASLD- and MASH-specific enhancements to the platform.

### Statistics

The sample size needed to evaluate the primary endpoint was informed using data reported in the Koutoukidis et al.^28^ review summarizing the impact of behavioral weight loss interventions on steatosis in MASLD. Using these results, it was determined a sample size of approximately 8 participants would provide approximately 90% power (α = 0.05) to detect −2.4% (SD −16) change in steatosis. To account for a 20% attrition rate, a minimum of 10 participants were required. This number was doubled based on the assumption that approximately 50% of those who met screening CAP >274 dB/m would not have an elevated baseline PDFF >10%.

All study endpoints and analyses were prospectively defined in the study protocol and/or the statistical analysis plan documents. SAS version 9.4 (Cary, NC) was used to perform the statistical analyses. A full statistical analysis plan documenting all data handling rules and prospective statistical analyses was finalized prior to completion of participant enrollment.

The baseline results were defined as the last assessment prior to PDT onboarding. The change from baseline was tested using a one-sample *t-*test if the normality assumption was met. The normality assumption was assessed using the Shapiro-Wilks test for normality. If the normality assumption was not met, then the change from baseline was tested using a Wilcoxon signed rank test. The authors had access to the study data and reviewed and approved the final manuscript.

## Results

### Study Population Characteristics

**Figure 1:**
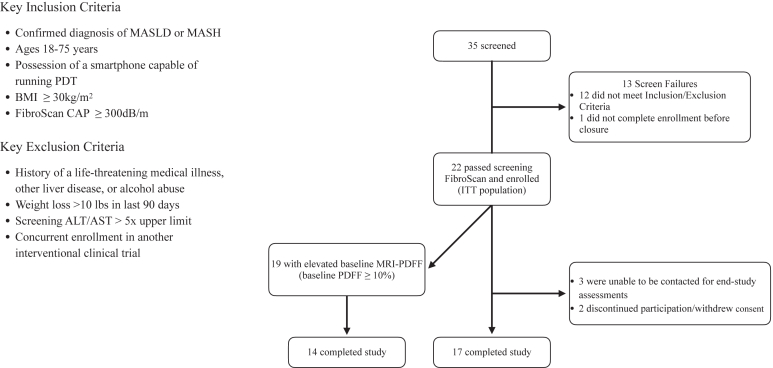
Study flow diagram and key eligibility criteria.

Baseline characteristics of the study population are presented in Table 1. The mean age (±SD) was 47.9 years (±14.1), 77% were female, the mean body mass index was 38.2 kg/m^2^ (±6.3), and 46% had type 2 diabetes. Participants had an average of 6.4 (±4.3) comorbidities and were taking an average of 6.2 (±4.1) medications at baseline, per medical history in electronic health records. The mean baseline MRI-PDFF liver fat content was 18.7% (±10.8) and the mean CAP score was 334.1 dB/m (±19.8). A total of 35 participants were screened for participation, 22 were enrolled, and 17 completed all baseline and postintervention measurements (Figure 1).

**Table 1:** Baseline Demographics in Safety Population

Parameter/Category	Safety population (n = 22)
Age, mean (SD)	47.9 y (14.1)
Sex, n (%)	
Female	17 (77)
Male	5 (23)
Ethnicity, n (%)	
Caucasian	9 (41)
Hispanic/Latino	10 (46)
Black	1 (5)
Mixed^a^	2 (9)
Body mass index, mean (SD)	38.2 kg/m^2^ (6.3)
Liver disease diagnosis at baseline	
MASH, n (%)	17 (77%)
MASLD, n (%)	5 (23%)
Liver fat (MRI-PDFF), mean (SD)	18.7% (10.8)
Controlled attenuation parameter (CAP) score^b^, mean (SD)	334.1 dB/m (19.8)
Liver stiffness measurement^b^, mean (SD)	8.2 kPA (2.7)
Fast™ score^b^, mean (SD)	0.39 (0.25)
Number of comorbidities, mean (SD)	6.4 (4.3)
Type 2 diabetes, n (%)	10 (46)
Hypertension, n (%)	13 (59)
Hyperlipidemia, n (%)	12 (55)
Number of concomitant medications, mean (SD)	6.2 (±4.1)

SD, standard deviation.

a Participant reported two or more ethnicities.

b As measured by FibroScan.

### Efficacy Endpoints

**Figure 2:**
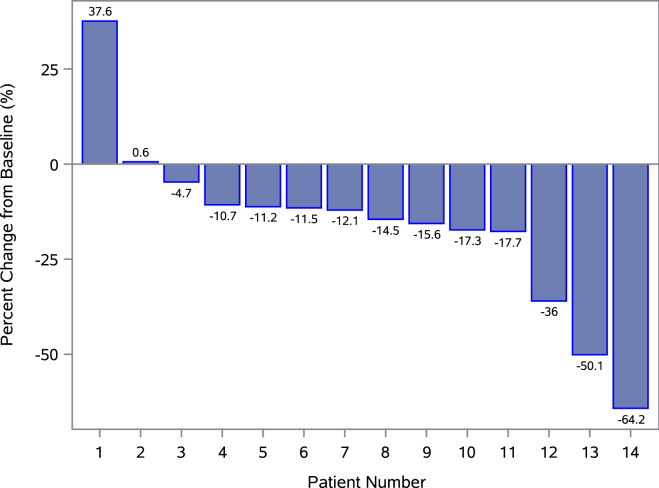
Percent change from baseline in liver fat by MRI-PDFF in participants with an elevated baseline. Waterfall plot shows change from baseline in MRI-PDFF for participants with a baseline PDFF ≥10% (n = 14). A mean change of −16.2% (*P* = .011) was observed using a one-sample Wilcoxon rank sum test.

Study outcomes are presented in Table 2. After 90 days of exposure to the PDT, the mean relative change in PDFF was −16.2% (±23.6, *P* = .011) in the 14 participants with baseline PDFF ≥10% (Figure 2). The maximum change observed within a single participant was a −64.2% relative reduction in PDFF from baseline.

**Table 2:** Change From Baseline in Study Endpoints

Parameter	Baseline (SD)	Mean absolute change (SD)	*P* value Mean absolute change	Mean percent change (SD)	*P* value Mean percent change
MRI-PDFF, %					
ITT population (n = 17)^a^	17.8 (10.7)	−2.2 (3.3)	*P* = .007	−13.2	*P* = .007
Baseline PDFF ≥10% (n = 14)^a^	20.6 (9.6)	−2.7 (3.4)	*P* = .013	−16.2	*P* = .011
FibroScan CAP score, dB/m					
ITT population (n = 17)	334.1 (19.8)	−18.8 (30.2)	*P* = .021	−5.7 (9.1)	*P* = .019
Baseline PDFF ≥10% (n = 14)	336.5 (20.5)	−16.7 (27.9)	*P* = .043	−5.0 (8.2)	*P* = .040
Liver stiffness measurement, kPA					
ITT population (n = 17)^a^	8.22 (2.7)	0.07 (3.0)	*P* = .411	−0.94 (31.8)	*P* = .404
Baseline PDFF ≥10% (n = 14)^a^	8.45 (2.8)	−0.43 (1.9)	*P* = .420	−5.31 (21.8)	*P* = .340
Fast™ score (%) (n = 17)^a^	0.39 (0.25)	−0.13 (0.16)	*P* = .006	−20.4 (71.1)	*P* = .011
ALT (IU/L)^b^					
ITT population (n = 17)	48.6 (31.4)	−17.1 (18.6)	*P* = .002	−24.7 (35.0)	*P* = .004
Elevated baseline ALT (n = 13)	59.0 (28.6)	−22.5 (17.6)	*P* = .001	−34.6 (19.3)	*P* = <0.001
FIB-4 (n = 17)^a^	1.1 (0.5)	−0.2 (0.3)	*P* = .051	−13.4 (19.8)	*P* = .045
Weight (lbs) (n = 17)^a^	236.0 (45.6)	−6.7 (−9.5)	*P* = .005	−2.9 (4.1)	*P* = .008

Parameter	Baseline (SD)	Mean absolute change (SD)	*P* value
Health-related quality of life (CDC-HRQOL) (n = 9)^a^	15.6 (11.1)	−2.2 (11.3)	*P* = .500

Parameter	Score
Net Promoter Score (n = 16)	+75.0
Promoter, %	81.3%
Passive, %	12.5/%
Detractor, %	6.3%

a Indicates *P* value obtained from a one sample Wilcoxon rank sum test. Otherwise, *P* value obtained from a one-sample *t*-test.

b Elevated ALT defined a priori as results > 19 IU/L in females and >30 IU/L in males.

**Figure 3:**
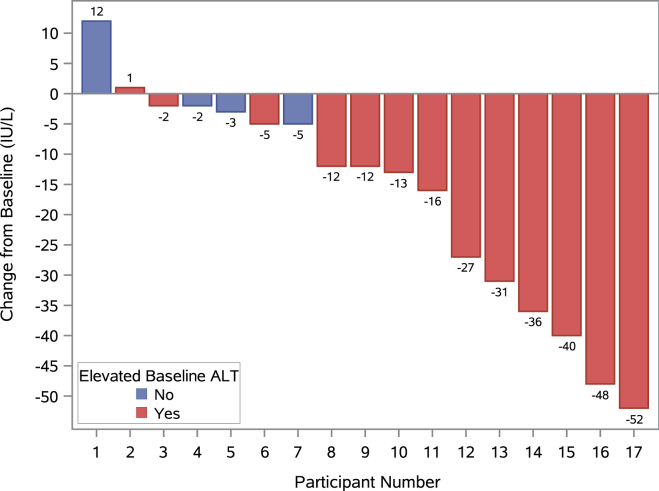
Change from baseline in alanine transaminase (ALT) in ITT population. Waterfall plot shows change from baseline in ALT for all participants in the ITT population (n = 17). A mean change of −17.1 IU/L (*P* = .002) was observed in the ITT population using a one-sample *t*-test. In those with an elevated ATL at baseline (n = 13), a mean change of −22.5 IU/L (*P* = .001) was observed using a one-sample *t*-test.

Figure 3 shows the change in ALT for each participant. The mean change in ALT was −17.1 IU/L (±18.6, n = 17, *P* = .002). In those with an elevated ALT at baseline (ALT >19 IU/L for females and >30 IU/L for males), the mean reduction was 22.5 IU/L (±17.6, n = 13, *P* = .001).

**Figure 4:**
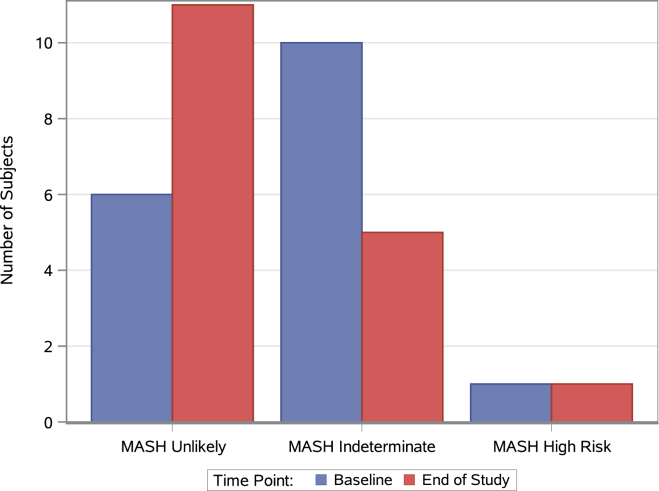
Change in Fast^TM^ score risk category. Comparison of the number of participants in the ITT population in each Fast^TM^ score risk category at baseline and postintervention.

CAP score was reduced by −18.8 dB/m (±30.2, n = 17, *P* = .021). Mean liver stiffness measurement was unchanged (increase of 0.07 kPA ±3.0, n = 17, *P* = .411), while an average relative reduction of −20.4% in the Fast™ score was observed (±71.1, n = 17, *P* = .011) with 45 % (5/11) of participants with a baseline Fast™ score in the high or indeterminate risk category moving to a lower risk category (Figure 4).

Participants achieved an average weight loss of −2.9% (±4.1, n = 17, *P* = .008) of total body weight, following a pattern of gradual and consistent weight loss without any signs of a plateau or peak (Figure A1).

### Adverse Events and Quality of Life Outcomes

A total of 10 adverse events were reported in 6 participants. Events were categorized as mild (n = 5) or moderate (n = 5) and none were related to the device. No serious adverse events or any device-related adverse events were reported (Table A1).

In participants who completed the baseline and end study HRQOL-4 Survey, there was an average improvement of +2.2 Healthy Days per month (±11.4, n = 9, *P* = .500). Participants also expressed a high degree of satisfaction with the PDT, with a mean NPS of +75 (n = 16).

### Engagement and Usability

Per protocol engagement with behavioral therapy was defined as completion of 10 weeks of CBT activities, including at least 10 lessons, 10 skills, and 10 weeks of goal-setting activities. The criteria were met by 15 of 22 participants in the intention-to-treat population. These participants spent an average of 48.1 minutes per week in the PDT and completed an average of 16.5 CBT lessons (±6.78) over the duration of the intervention. Ninety-four percent of participants were still using the app in week 12, the final week of the intervention.

A qualitative analysis was completed to identify themes from participant feedback about their experience. Predominate themes were that the PDT helped participants develop an increased sense of self-efficacy, knowledge of behavioral strategies to improve health, and sense of autonomy related to self-management of their condition. Of the participants who completed a postintervention qualitative interview (n = 16), all expressed a desire to continue using the PDT if given the option for a longer intervention period.

## Conclusion

This first-of-kind study of a PDT in patients with MASLD/MASH demonstrated improvements across multiple markers, including MRI-PDFF, FibroScan CAP score, and weight. Clinically meaningful improvements in Fast™ score and ALT were also observed indicating the clinical potential of this intervention in MASLD/MASH treatment in a larger patient population.

A gradual and consistent pattern of weight loss observed over the 90-day intervention is consistent with a CBT-based mechanism of action in which a person continuously learns, adopts, and maintains new patterns of behavior. The magnitude of weight loss observed was greater than what was observed in the recent type 2 diabetes pivotal trial using the same underlying CBT platform^24^ indicating a robust response to treatment in this patient population. The lack of a clear weight loss peak in weekly weight trends suggests that longer use of the PDT could result in continued weight loss and additional improvements in liver biomarkers.

Clinically meaningful outcomes were seen using a version of the CBT designed to improve cardiometabolic health in general. Enhancing the PDT with MASLD/MASH-specific content may improve the therapeutic potential in these conditions.

The safety data generated from this study were encouraging. The lack of device-related adverse events, despite the high use of background pharmacotherapy and multiple comorbidities, suggests a relatively low risk for this behavioral therapy when used in clinical practice. Importantly, given its behavioral mechanism of action, the PDT can be used in conjunction with future approved medications to enhance efficacy with potentially fewer adverse treatment interactions than can arise when combination drug strategies are employed.

Limitations of this study include the small, non-randomized, open-label study design and short-term follow-up. Furthermore, given the lack of control, this study cannot rule out the possibility of a Hawthorne effect. Participants were used as their own controls and the absence of a comparator arm limits the ability to make broader conclusions on efficacy and safety. For this reason, we plan to validate the results in a future larger randomized controlled trial.

Participants were recruited from 2 clinic locations in the same metropolitan area and therefore may not be representative of the broader domestic or international MASLD or MASH populations. Nonetheless, the findings from this initial proof-of-concept study in MASLD and MASH provide compelling evidence to shape the design of follow-up studies to further evaluate the potential of this PDT in a larger and control-matched population over a longer study duration. To our knowledge, this is the first study that uses a CBT-based digital therapeutic in MASLD/MASH. PDTs are a growing therapeutic sector and offer unique treatment opportunities beyond traditional pharmaceuticals. The Access to Prescription Digital Therapeutics Act,^29^ currently under legislative review by the United States Congress, aims to expand coverage of PDTs to the millions of Americans who are insured through Medicare and Medicaid programs. Such efforts indicate the potential for PDTs to provide accessible and cost-effective treatment options to patients at scale.

Strengths of this study include evaluating the use of the PDT within a clinical environment representative of its intended use, and inclusion of patients with multiple cardiometabolic comorbidities on robust background pharmacotherapy.

Digitally delivered behavioral therapies that are used at the patient’s convenience offer the greatest potential for scalability and accessibility of behavioral interventions. The addition of this PDT to providers’ toolkits may enable a more effective system of care that improves health equity without adding complexity to disease management responsibilities.

Patient satisfaction, a good indicator of acceptability and adherence, was very high as evidenced by an NPS of more than double the average satisfaction rating of +32 across health-care benchmarks.^30^ Patient engagement after 12 weeks was also 2.5 to 3 times higher than the average observed in other mobile health and wellness apps.^31^

The study demonstrated clear and consistent improvements in liver health in patients with MASLD and MASH in a broad set of validated biomarkers, including MRI-PDFF, FibroScan, weight, and several blood biomarkers. While further research is needed to establish peak effectiveness, the totality of positive efficacy, safety, and usability data observed in this feasibility study raises the potential this PDT may help address the significant unmet clinical and public health needs observed in MASLD/MASH by providing scalable, accessible, and effective behavioral therapy. There is great potential for a system of care where PDTs are prescribed as a first-line treatment or in conjunction with pharmacologic agents to enable patients to reach treatment goals and improve quality of life.

## Supplementary Materials

Study protocol

Table A1

Figure A1

Legend of Figure A1
