# Supplementary material for: A Novel Prescription Digital Therapeutic Option for the Treatment of Metabolic Dysfunction-Associated Steatotic Liver Disease
Source: Gastro Hep Adv. 2023 Oct 1;3(1):9–16. doi: 10.1016/j.gastha.2023.08.019 (PMC11307699; doi:10.1016/j.gastha.2023.08.019)
Supplement: Study protocol [file mmc1.pdf]

Research Study Protocol

BT-NCBT-00x: Nutritional Cognitive Behavioral Therapy Feasibility Study in NAFLD and NASH

(LivVita Study)

Protocol Number: BT-NCBT-00x

Principal Investigator: Dr Naim Alkhouri

Sponsor: Better Therapeutics, Inc.

Version: 1.1

Date: 18 April 2022

Protocol Approval Signature:

The signatory has reviewed and agrees to the content of the final clinical study protocol as presented.

**Name**	**Naim Alkhouri, MD**	**Date**
**Role:**	**Principal Investigator**	

**Name**	**Aleks Skuban, MD**	**Date**
**Role:**	**Sponsor Representative**	

Table of Contents

Protocol Synopsis

Background

Study Hypotheses

Outcomes

- Measures of Primary Outcomes
- Measures of Secondary Outcomes
- Exploratory Outcomes

Participant Population

- Inclusion Criteria
- Exclusion Criteria

Study Procedures

- Recruitment and Screening
- Informed Consent Process
- Study Enrollment
- Behavioral Intervention
- Study Measurements
- Table 1: Schedule of Assessments

Adverse Event Reporting

- Definition of Adverse Events and Serious Adverse Events
- Events Meeting the AE Definition
- Events Not Meeting the AE Definition
- Events Meeting the Serious Adverse Event (SAE) Definition
- Adverse Event Severity
- Relationship to Study Intervention
- Serious Adverse Event Reporting
- Following up on Adverse Events
- Adverse Device Effects (ADEs)
- Documenting ADEs
- Follow-up of ADEs
- ADE Reporting

Participant Compensation

Study Terminations

Sample Size

Analysis Populations

Statistical Analysis

Primary and Secondary Endpoint Analyses

Exploratory Endpoint Analyses

Safety Summaries

Data Storage

Risks and Benefits

Ethical Guidelines and Institutional Review Board

References

Appendices

Appendix 17.1: Study Enrollment Landing Page

Appendix 17.2: BT-NCBT-00x Digital Therapeutic (Daily Use)

Appendix 17.3: Behavioral Support Outreach and Scheduling

Appendix 17.4: BT-NCBT-00x Agnostic Treatment Experience Survey

Appendix 17.5: Health Days Core Module (CDC HRQOL-4)

Appendix 17.6: Net Performance Score (NPS)

Protocol Synopsis v1.0

**Title**	**Nutritional Cognitive Behavioral Therapy Feasibility Study in NAFLD and NASH**<br><br>**LivVita Study**
**Clinical Study Type**	Single arm interventional cohort study
**Name of Device**	Better Therapeutics’ Nutritional Cognitive Behavioral Therapy (BT-NCBT-00x)
**Sponsor**	Better Therapeutics, Inc<br>548 Market Street, #49404<br>San Francisco, CA 94104
**Indication under investigation**	Better Therapeutics’ BT-NCBT-00x is a software program intended to help patients with NASH/NAFLD, under the guidance of their physician, improve biometric markers. The BT-NCBT-00x software delivers nutritional cognitive behavioral therapy to patients via a mobile application (App) that targets behaviors related to improving cardiometabolic disease.
**Objective(s)**	The primary objective is to evaluate the feasibility and efficacy of BT-NCBT-00x in improving liver health in patients diagnosed with nonalcoholic fatty liver disease (NAFLD) or nonalcoholic steatosis (NASH).<br><br>The secondary objective is to gather user-experience feedback that will be used to improve usability of BT-NCBT-00x for future use in patients with NAFLD/NASH and evaluate intervention safety in this patient population.<br><br>In addition, this study will explore the degree to which various non-invasive imaging technologies, composite scores, and serum laboratory biomarkers are sensitive to behavioral changes induced by BT-NCBT-00x.

**Primary Endpoint**	1) Mean change in percent liver fat from baseline to end of treatment in participants with baseline PDFF ≥ 10%, as measured by MRI-PDFF.
**Secondary Endpoints**	1) Mean change in liver fat from baseline to end of treatment in all participants, as measured by MRI-PDFF<br>2) Percent of participants who achieve ≥ 30% reduction in PDFF from baseline to end of treatment
**Exploratory Endpoints**	1) Mean change in liver fat, as measured by Fibroscan Controlled Attenuation Parameter (CAP) score<br>2) Percentage of participants who achieve > 20dB/m reduction in CAP score<br>3) Mean change in liver stiffness as measured by Fibroscan Vibration Controlled Transient Elastography in kPA<br>4) Percentage of participants who achieve > 20% relative reduction in liver stiffness from baseline, measured by kPA<br>5) Mean percent change in total body weight<br>6) Mean change in ALT<br>7) Percentage of participants who achieve > 17IU/L reduction in ALT<br>8) Patient health-related quality of life score as measured by CDC HRQOL-4, *dispensed in app*<br>9) Net Promoter Score (NPS), *dispensed in app*<br>10) Mean percent change in FAST score<br>11) Mean change in HbA1c<br>12) Change in NASH risk from baseline to end of intervention, as measured by change in FAST score categories (> 0.67 = high risk for NASH, 0.67-0.35 indeterminate, < 0.35 = NASH is unlikely)<br>13) Qualitative feedback on user experience, collected via in-App Treatment Experience Survey and participant calls with the study sponsor's support team

**Enrollment Criteria**	***Inclusion Criteria***<br>Participants who meet the following criteria will be eligible to participate in the study:<br>1) Male and female patients between 18 and 75 years old, inclusive, at the time of signing the informed consent;<br>2) Diagnosis of NAFLD or NASH, per medical record;<br>3) At least 2 doses of COVID-19 vaccination, per self-report;<br>4) Possession of a smartphone (iPhone or Android only) capable of running the BT-NCBT-00x smartphone application (App) used in the study, assessed during the screening visit;<br>5) BMI ≥30 kg/m^2 at the screening visit;<br>6) Fibroscan CAP ≥ 300 dB/m collected at screening assessment;<br>7) Completion of baseline MRI-PDFF;<br>8) Capable of giving informed consent in English, which includes compliance with the requirements and restrictions listed in the informed consent form (ICF) and in this protocol.<br>***Exclusion Criteria***<br>Participants will not be eligible to participate if they meet any of the following criteria:<br>1) Are unable to understand, consent to, or comply with the study protocol for any reason, including the inability to read or comprehend English. The behavioral intervention mechanisms, including educational materials and digital tools, are only available in English at this time;<br>2) Documented medical history or self-report of:<br>a) An unstable or life-threatening medical illness, precluding full compliance with the study protocol (e.g., emergency room visit or hospitalization within the prior 30 days; cancer not in remission; moderately-severe or severe heart failure; severe or end-stage kidney failure; alcoholic fatty liver disease or cirrhosis; active suicidality or psychotic disorder);<br>b) Weight loss of greater than 10 pounds within the last 90 days prior to screening visit;

	c) For women only: pregnant (or lactating) or having the intention of becoming pregnant during the time frame of the study. Pregnant and lactating women are subject to weight gain or loss that differentiates them from a non-pregnant or lactating population;<br>3) Concurrent enrollment in any other interventional clinical trial; less than 30 days from last intervention of dose of medication in any other research study for any therapeutic indication;<br>4) Presumed or confirmed COVID-19 diagnosis within 30 days prior to study enrollment;<br>5) Is considered unreliable by the investigator, or having any condition that, in the opinion of the investigator, would not allow safe participation in the study;<br>6) Change in medication regimen of hormonal contraceptives, anti-hyperglycemic medications, medications for mental or emotional disorders, corticosteroids, thyroid hormones, Tamoxifen, or Methotrexate within 90 days of before study screening;<br>7) ALT or AST > 5 times the upper normal limit at baseline;<br>8) History of other forms of liver disease, including hepatitis-A, hepatitis-B, hepatitis-C, or Wilson's disease, primary biliary cholangitis, primary sclerosing cholangitis, autoimmune hepatitis, Alpha-1 antitrypsin deficiency; or history of HIV;<br>9) Recent history of (within 12 months of treatment initiation) or strong potential for alcohol or substance abuse. Alcohol abuse defined as >21 drinks per week for male and >14 drinks per week for female (1 drink = 12 oz beer, 5 oz wine, or 11⁄2 oz distilled spirits).
**Study Design**	This single arm interventional cohort study is designed to explore the feasibility of using BT-NCBT-00x to improve liver fat, inflammation, and stiffness in patients diagnosed with NAFLD or NASH over a 3-month intervention.<br><br>Potential study participants will be identified by PI and designated study team at Arizona Liver Health during clinic visits. Eligible

	participants will be screened and consented by the research team, per the procedures listed below. FibroScan will be used to screen individuals likely to meet the PDFF primary endpoint analysis criteria of a liver fat fraction greater than or equal to 10% at baseline. All participants will be included in secondary and exploratory endpoint analysis.<br><br>Labs and imaging will be collected at screening visit to confirm eligibility requirements and establish a baseline, then again at the end of the intervention to assess therapeutic impact. Participants will also be asked to complete qualitative assessments to evaluate their experience using the study intervention and the impact of treatment on their quality of life.<br><br>The full Schedule of Assessments is provided in Table 1 within the protocol.<br><br>The treatment, BT-NCBT-00x, consists of a software as a medical device developed by the study Sponsor, Better Therapeutics. It delivers treatment to participants with cardiometabolic disease, using behavioral therapy that targets individual behaviors related to improving dietary quality and physical activity. BT-NCBT-00x is accessed via the participants' smartphone after downloading from the phone's corresponding app store.<br><br>Participants will receive behavioral support by the Better Therapeutics patient services team in the form of an orientation and wrap-up call. The intent of the Treatment Orientation Call is to introduce participants to the nature of the treatment and assess readiness to engage in behavioral therapy. In this call, participants will also receive education on evidence-based behavioral strategies for NAFLD treatment that are not yet fully integrated into BT-NCBT-00x, such as the benefit of reducing intake dietary saturated fat and replacing with monounsaturated and polyunsaturated fatty acids. The wrap-up session is intended to allow the Better Therapeutics team to gather feedback about participants' experiences using the intervention. The study population will also be monitored by the Better Therapeutics Population Health Nurse Practitioner and participants will be offered additional behavioral

	support if they are not meeting biometric or engagement progress metrics.<br><br>Participants will continue their usual medical care for their condition(s) and utilize BT-NCBT-00x for nutritional cognitive behavioral therapy for one 90-day treatment cycle.
**Screening Procedures & Schedule of Assessments**	1) Participants are pre-screened for a diagnosis of NAFLD or NASH, per medical record<br>2) Participants are screened for interest in study participation & eligibility.<br>3) After providing informed consent, participant completes initial FibroScan screening (CAP < 300dB/m is a screen failure).<br>4) Participant completes collection of screening labs, biometrics, and focused physical exam and is scheduled for MRI-PDFF and Treatment Orientation Call.<br>5) The participant will complete a Treatment Orientation Call with a Better Therapeutics' Product Support Specialist prior to using BT-NCBT-00x.<br>6) Participant completes MRI-PDFF.<br>7) PI reviews all data to confirm participant eligibility.<br>8) Participants who meet all inclusion and exclusion criteria will then be referred for initiation of treatment through the Better Therapeutics Study Portal.<br>a) Referral requires study staff to enter the patient's unique ID, mobile phone number, and email into the Better Therapeutics Study Portal.<br>9) The participant receives an email and text with a link to create an account and download the BT-NCBT-00x App.<br>a) Participants who do not complete App onboarding will be considered screen failures and will be replaced.<br>10) The participant completes 90-day behavioral intervention using BT-NCBT-00x.<br>11) The participant returns to clinic post-study visit for repeat Fibroscan, labs, biometrics and MRI-PDFF.

**Study Population**	The study population consists of approximately 20 male and female adult participants (18 – 75 years old), with a diagnosis of nonalcoholic fatty liver disease (NAFLD) or nonalcoholic steatohepatitis (NASH), and who satisfy the inclusion and exclusion criteria.
**Study Sites**	Two clinic sites at Arizona Liver Health, located in Chandler and Peoria, Arizona.
**Compensation**	All participants will be compensated for participation based on completion of study milestones depending on study activities completed.
**Safety Assessments**	Subjects will be assessed for Serious Adverse Events (SAEs) throughout the study starting from the time of signing the informed consent form. Subjects will be assessed for Adverse Events (AEs) from the time of study intervention.<br>Occurrence, relatedness, and severity of all SAEs and AEs will be evaluated and summarized at the end of the 3-month study period by the Principal Investigator.<br>All SAEs shall be reported to the Sponsor within 24 hours of notice of SAE.
**Statistics**	<u>Sample Size</u><br>Sample size calculations are estimated for the primary analysis population, i.e. those who satisfy all inclusion/exclusion criteria and have a baseline PDFF ≥ 10%.<br>The sample size was calculated by referencing the mean absolute change in steatosis and standard deviation reported in a 2019 JAMA review of the impact to behavioral weight loss interventions on clinical markers of NAFLD (Koutoukidis et al., 2019). The average mean absolute change in steatosis was -2.4 and mean standard deviation was 1.6.

	Using these results, it was determined a sample size of approximately 8 participants would provide approximately 90% power, assuming an alpha of 0.05. To account for up to a 20% attrition rate, a minimum of 10 participants are required.<br><br>To ensure inclusion of a mix of patients with either NAFLD or NASH, a total of approximately 20 participants will be enrolled. It's expected that up to 50% of fully eligible participants will fail to meet the MRI-PDFF requirements for inclusion in the primary endpoint analysis, resulting in approximately 10 participants included in the primary endpoint analysis. Participants who do not meet the MRI-PDFF requirement for primary analysis endpoint will be included in secondary and exploratory analyses.<br><br>Demographics and baseline characteristics will be summarized descriptively for all subjects based on the analysis population. Descriptive summaries will include the number of non-missing observations, mean, median, standard deviation, minimum and maximum for continuous results. Categorical results will be summarized with counts and percentages.<br><br><u>Efficacy Analyses</u><br><br>The primary efficacy endpoint is the change in percentage liver fat as measured by MRI-PDFF from baseline to 3 months after enrollment in those with a baseline PDFF ≥ 10%. The results at baseline, at 3 months post-enrollment, and the change from baseline will be summarized descriptively. In addition, the change from baseline in the percentage liver fat will be tested using a one-sample t-test if the normality assumption is met. If the normality assumption is not met, then the change from baseline will be tested using a Wilcoxon rank sum test.<br><br>The continuous secondary and exploratory endpoints will be summarized in the same manner as described for the primary efficacy endpoint.<br><br>The categorical secondary and exploratory endpoints, such as the percentage of participants who achieve ≥ 30% reduction in PDFF from baseline to post-intervention, will be summarized using counts

	and percentages. In addition, exact binomial confidence intervals for the true rates will be provided.<br><br>Safety Summaries<br>Treatment-emergent Adverse Events will be summarized using counts and percentages. A treatment-emergent adverse event (TEAE) will be defined as any AE that occurs after the start of use of the Better Therapeutics App. Summaries of AEs will include:<br><br>● All TEAEs<br>● Serious TEAEs<br>● TEAEs by severity and relationship to treatment<br>● and medical software incidents
**Total Study Duration**	Each enrolled participant will be exposed to a 90-day treatment intervention. The treatment begins from the day the participant downloads the App and completes onboarding and continues for the subsequent 90 days. The imaging required to evaluate primary endpoints must be completed within 14 days following completion of the intervention.

1. Background

Nonalcoholic fatty liver disease (NAFLD) is a growing threat to public health. The condition currently affects an estimated 20-30% of all US adults (Cotter & Rinella, 2020; Younossi et al., 2018) and approximately 70% of those with type two diabetes (Younossi et al., 2019). The more advanced form of this condition, called nonalcoholic steatohepatitis (NASH), affects approximately 5% of American adults and has recently become the leading indication for liver transplant (Cotter & Rinella, 2020; Younossi et al., 2018).

Despite the magnitude of these conditions, no FDA-approved treatments currently exist. Behavioral modification including weight loss, improving dietary quality and increasing physical activity have been proven have favorable effects on slowing and even reversing the progression of liver steatosis and fibrosis (Johnson et al., 2009; Polyzos et al., 2019; Younossi et al., 2021), however, behavior change is notoriously difficult to facilitate in clinical practice. Many providers struggle to provide effective behavioral counseling due to lack of knowledge, time, and resources and are unable to help patients implement targeted, personalized behavioral therapy to improve NAFLD or NASH (Hallsworth & Adams, 2019).

Better Therapeutics has created a novel digital therapeutic platform for the treatment of cardiometabolic disease using nutritional cognitive behavioral therapy (N-CBT). The treatment has been associated with clinically meaningful outcomes in type 2 diabetes (Berman et al., 2018; Guthrie et al., 2020) and hypertension (Guthrie et al., 2019), both of which have similar underlying metabolic pathways to NAFLD and NASH. It is believed there is therapeutic potential for the Better platform to be used in patients with NAFLD and/or NASH as an effective treatment option. This feasibility study is designed to explore efficacy, usability and safety of this novel therapeutic to assess its potential as a future treatment option for these conditions and to help plan future studies.

2. Study Hypotheses

The primary hypothesis is that participants using a digital therapeutic delivering behavioral therapy will achieve meaningful reductions in steatosis after 3 months when compared to baseline. Additional hypotheses are that participants will achieve improvements in other markers of liver health.

3. Outcomes

3.1 Measures of Primary Outcomes

- Mean change in percent liver fat from baseline to end of treatment in participants with baseline PDFF ≥ 10%, as measured by MRI-PDFF

3.2 Measures of Secondary Outcomes

All secondary outcomes will be assessed at baseline and 3 months after enrollment. The secondary outcomes are:

- Mean change in liver fat from baseline to end of treatment in all participants, as measured by MRI-PDFF
- Percent of participants who achieve ≥ 30% reduction in PDFF from baseline to post-intervention

3.3 Exploratory Outcomes

All exploratory outcomes will be assessed at baseline and approximately 90-days months after enrollment. The exploratory outcomes are:

- Mean change in liver fat, as measured by Fibroscan Controlled Attenuation Parameter (CAP) score
- Percentage of participants who achieve > 20dB/m reduction in CAP score
- Mean change in liver stiffness as measured by Fibroscan Vibration Controlled Transient Elastography in kPA
- Percentage of participants who achieve > 20% relative reduction in liver stiffness from baseline, measured by kPA
- Mean percent change in total body weight
- Mean change in ALT
- Percentage of participants who achieve > 17IU/L reduction in ALT
- Patient health-related quality of life score as measured by CDC HRQOL-4, *dispensed in app*
- Net Promoter Score (NPS), *dispensed in app*
- Mean percent change in FAST score
- Change in NASH risk, as measured by change in FAST score categories (> 0.67 = high risk for NASH, 0.67-0.35 indeterminate, < 0.35 = NASH is unlikely)
- Qualitative feedback on user experience, collected via in-App Treatment Experience Survey and participant calls with the study sponsor's support team

4. Participant Population

The study population consists of approximately 20 male and female adult participants (18 – 75 years old), with a diagnosis of nonalcoholic fatty liver disease (NAFLD) or nonalcoholic steatohepatitis (NASH), and who satisfy the inclusion and exclusion criteria.

4.1 Inclusion Criteria

Participants who meet the following criteria will be eligible to participate in the study:

1) Between 18 and 75 years old, inclusive, at the time of signing the informed consent;
2) Diagnosis of NAFLD or NASH, per medical record;
3) At least 2 doses of COVID-19 vaccination, per self-report;
4) Possession of a smartphone (iPhone or Android only) capable of running the BT-NCBT-00x smartphone application (App) used in the study;
5) BMI ≥30 kg/m^2 recorded at screening visit;
6) Fibroscan CAP ≥ 300 dB/m collected at screening visit;
7) Completion of baseline MRI-PDFF (only participants with liver fat fraction values ≥10% will be included in primary analysis);
8) Capable of giving informed consent in English, which includes compliance with the requirements and restrictions listed in the informed consent form (ICF) and in this protocol.

4.2 Exclusion Criteria

Participants <u>will not</u> be eligible to participate if they meet any of the following criteria:

1) Are unable to understand, consent to, or comply with the study protocol for any reason, including the inability to read or comprehend English. The behavioral intervention mechanisms, including educational materials and digital tools, are only available in English at this time;
2) Medical history or self-report of:
    a) An unstable or life-threatening medical illness, precluding full compliance with the study protocol (e.g., emergency room visit or hospitalization within the prior 30 days; cancer not in remission; moderately-severe or severe heart failure; severe or end-stage kidney failure; alcoholic fatty liver disease or cirrhosis; active suicidality or psychotic disorder);
    b) Weight loss of greater than 10 pounds within the last 90 days prior to study enrollment;

c) For women only: pregnant (or lactating) or having the intention of becoming pregnant during the time frame of the study. Pregnant and lactating women are subject to weight gain or loss that differentiates them from a non-pregnant or lactating population;

3) Concurrent enrollment in any other interventional clinical trial; less than 30 days from last intervention of dose of medication in any other research study for any therapeutic indication;
4) Medical history or self-report of COVID-19 diagnosis within 30 days prior to study enrollment;
5) Is considered unreliable by the investigator, or having any condition that, in the opinion of the investigator, would not allow safe participation in the study;
6) Change in dose or quantity of hormonal contraceptives, anti-hypertensive medication, anti-hyperglycemic medications, medications for mental or emotional disorders, corticosteroids, thyroid hormones
7) Current use of tamoxifen or methotrexate
8) ALT or AST > 5 times the upper normal limit at baseline;
9) History of other forms of liver disease, including hepatitis-A, hepatitis-B, hepatitis-C, or Wilsons disease, primary biliary cholangitis, primary sclerosing cholangitis, autoimmune hepatitis, Alpha-1 antitrypsin deficiency; or history of HIV;
10) Recent history of (within 12 mo of Day -7) or strong potential for alcohol or substance abuse. Alcohol abuse defined as >21 drinks per week for male and >14 drinks per week for female (1 drink = 12 oz beer, 5 oz wine, or 11⁄2 oz distilled spirits).

5. Study Procedures

5.1 Recruitment & Screening

Arizona Liver Health Clinics will utilize their patient network to identify eligible patients and additional IRB approved recruitment methods may be utilized for patient recruitment. Most individuals are expected to be identified by providers during clinic visits who have a historic diagnosis of NAFLD or NASH. Patients are screened for interest in study participation. If interested, participants will be screened and guided through the informed consent process by a member of the research staff (see below). After providing informed consent, patients will receive a screening FibroScan. A CAP score < 300dB/m is considered a screen failure. Participants then complete a collection of baseline labs, biometrics, focused physical exam, BMI measurement, Treatment Orientation Call, and MRI-PDFF. PI reviews all data to determine participant eligibility for primary or secondary analysis groups.

5.2 Informed Consent Process

A properly executed informed consent will be obtained from each participant prior to any study-specific activities being completed. Delegated staff at the study site will facilitate the informed consent process with eligible participants. Potential participants will have the option of contacting study personnel with any questions or concerns before providing a signature on the consent. Participants will be provided a physical copy of their signed consent form. Participants will be able to withdraw from the study at any time by emailing or calling study personnel using the contact information provided in this form.

5.3 Study Enrollment

Participants who are consented and eligible for participation will be referred by the study site to begin treatment with the BT-NCBT-00x study App. Referral requires clinic staff to enter the patient's unique ID, mobile phone number, and email into the Better Therapeutics Study Portal. The portal is secure and HIPAA compliant.

Once referred, the participant will receive an email and text with link directing them to a secure website to create an account for the study intervention (see Appendix 17.1). Account creation will require participants to provide an email address, mobile phone number, first name and last name. This information will be validated against the values added to the Study Portal by the Arizona Liver Health delegated staff to confirm the App is being downloaded by the correct individual. The creation of the account triggers an SMS with a link to download the App from the app store appropriate to the phone type. Enrollment in the study will be completed once a participant completes the self-directed onboarding within the App. Participants who do not complete App-onboarding will be considered screen-failures and replaced.

5.4 Behavioral Intervention

BT-NCBT-00x was created by the Sponsor, Better Therapeutics, Inc. It delivers treatment to participants with cardiometabolic disease, using behavioral therapy that targets individuals' beliefs and behaviors related to improving dietary quality and physical activity. Sample screen images of digital therapeutic can be seen in Appendix 17.2.

BT-NCBT-00x is accessed via the participants' smartphone after downloading from the phone's corresponding app store. The mobile app that study participants will download for the study is called "Better" in the app stores.

The behavioral intervention process involves: 1) identifying maladaptive thoughts based on misinformed or false underlying core beliefs (e.g., those related to macronutrient fears, the hedonic nature of eating, physical exertion, other perceived barriers to changing lifestyle) that

lead to disease-promoting behaviors; 2) replacing these maladaptive core beliefs and thought patterns with adaptive ways of thinking developed from rational reflection; 3) providing collaborative (between participant and device) construction of behavioral exercises to test core beliefs; and 4) using additional validated behavioral techniques to enhance a participant's capacity to solve problems, plan activities and cope with interfering emotions or thoughts. BT-NCBT-00x helps participants understand the steps they should prioritize by presenting them with a treatment plan that summarizes their daily and weekly goals.

Each week, BT-NCBT-00x asks participants to complete a new behavioral module, along with one or more skill-based exercises that are related to that week's module. All participants are exposed to the same course of modules. The modules are expected to take between 10-20 minutes to complete. The modules are called "lessons" within BT-NCBT-00x. Each lesson addresses core beliefs in one of the following areas:

- Personal beliefs and barriers, such as those related to a participant's ability to change and control his or her behaviors;
- Beliefs about macronutrients and the importance of various food types;
- Hedonic-related beliefs about pleasant or unpleasant sensations experienced by eating or exercising; and
- Beliefs about exercise.

Skill exercises are intended to provide an opportunity for participants to practice skills related to behavioral beliefs. The method by which participants must practice these skills is designed to enhance executive function tasks such as planning, problem solving and goal setting. Each lesson explains the rationale and benefits of the skill exercise in reference to the core topic being explored.

In addition to completing a lesson (and one or more skill exercises), BT-NCBT-00x directs patients to self-report diet and exercise behaviors, medication adherence, and biometrics (e.g., weight). If the patient has comorbid Type 2 diabetes or hypertension, he or she will also be able to self-monitor fasting blood glucose and blood pressure each day. Weekly behavioral goals, including diet and exercise behaviors, are determined through a goal setting exercise, and are advanced in a manner most likely to maintain or increase self-efficacy. When self-efficacy is high, the participant has a high probability of achieving his or her goals.

Participants will be directed to engage with one lesson each week of the study, to report plant-based meals consumed and minutes of exercise completed and, if indicated, to measure

their blood glucose and/or blood pressure daily. All participants will be given access to the App for 3-months.

5.5 Product Support

A selection of patient services will be provided over the course of the study. Participants will be expected to complete two calls with a behavioral specialist: the Treatment Orientation Call will be completed prior to the start of intervention and a wrap-up call completed at the end of the intervention. The Treatment Orientation Call is intended to help introduce the participant to the nature of the treatment and assess readiness to engage in behavioral therapy. In this call, participants will also receive education on evidence-based behavioral strategies for NAFLD treatment that are not yet fully integrated into BT-NCBT-00x, such as the benefit of reducing dietary intake of saturated fat and replacing with monounsaturated and polyunsaturated fatty acids. The wrap-up session is intended to allow the Better Therapeutics team to gather feedback about participants' experiences using the intervention.

The study population will also be monitored by a Nurse Practitioner to assess participants' engagement and biometric progress. Users who are not meeting expected thresholds for engagement or biometric progress will be offered additional support in the form of a call with a behavioral specialist or nurse practitioner to help identify barriers to behavioral therapy and develop a plan for addressing these potential obstacles to improve treatment adherence and efficacy. Sample screen images of support messages and call scheduling ability in the App can be seen in Appendix 17.3.

A product support specialist will be available over the length of the study to provide support for participants who need help using BT-NCBT-00x. If a participant's needs are beyond the scope of technical product support, the product support specialist will refer the participant to the clinical care team for behavioral support. In addition, a behavioral support specialist may proactively reach out to patients to offer more support to help them get the most out of using BT-NCBT-OOX. Participants will be free to accept or turn down any offer of support.

5.6 Study Measurements

Treatment Experience Survey. A set of 8 questions asking about a participant's experience using the App (BT-NCBT-00x) will be dispensed on day 80 after enrollment within the App. The survey questions can be seen in Appendix 17.3.

Healthy Days Measure. The standard set of 4-items of the Healthy Days core questions (CDC HRQOL-04) will be dispensed within the App at day 1 and at treatment day 76. The survey questions can be seen in Appendix 17.4.

Net Promoter Score (NPS). Participants will be asked to complete an NPS survey on day 85 within the App. The NPS is a standardized tool for measuring a participant's overall satisfaction with the program. Participants will be asked how likely they are to recommend the app to a friend with a relevant condition and will be asked to enter a number from 1 to 10. The NPS tool can be seen in Appendix 17.5.

Table 1: Schedule of Assessments

**Procedures**	**Pre-enrollment Screening**	**Study Intervention**<br><br>**D1 to D90**	**Post-Intervention Study Visit**<br><br>**D90 to D104**
Patients identified with a history of NAFLD or NASH	X[1]		
Informed consent	X		
Labs, biometrics, focused physical exam, BMI	X		X
Fibroscan >300 db/m	X		X
Treatment Orientation Call scheduled	X		
MRI-PDFF (to be done after Fibroscan)	X[2]		X
Review & confirmation of participant eligibility by PI	X		X
Research staff refers eligible participants using Study Portal	X		
AE Reporting	X[3]	X[4]	X[4]
Installation of App & completion of first-time user experience		X[5]	

Study staff contacts patient to schedule post-treatment visit & MRI		X[6]	
Healthy Days Measure *(HRQOL-04)*		X[7] Day 1 and 76	
Net Promotor Score		X[7] Day 85	
Treatment Experience Survey		X[7] Day 80	

X[1] - Patients may be identified during clinic visits, through pre-screening in patient database, or other IRB approved recruitment methods
X[2] - MRI must be completed prior to patient being referred to begin treatment
X[3] - Assess and document serious adverse events
X[4]- Assess and document serious adverse events and adverse events
X[5] - Participant's completion of onboarding and first time user experience marks treatment Day 1
X[6] – This will begin on Day 74
X[7]- Surveys are automatically dispensed to participants in-App on designated days

Color Key:
= These activities occur during the screening visit
= These activities are conducted within the study-App and require no research staff involvement.

6. Adverse Event Reporting

6.1 Definition of Adverse Events and Serious Adverse Events

AEs will be assessed throughout the study. SAEs will be assessed from the time of signing the informed consent form and AEs will be assessed starting at the time of starting the intervention.

An AE is any untoward medical occurrence in a participant or clinical study participant, temporally associated with the use of study intervention, whether or not considered related to the study intervention. An AE can therefore be any unfavorable and unintended sign (including an abnormal laboratory finding), symptom, or disease (new or exacerbated) temporally associated with the use of study intervention.

6.2 Events Meeting the AE Definition

- Any abnormal laboratory test results (e.g., hematology, clinical chemistry, or urinalysis) or other abnormal safety assessments (e.g., ECG, radiological scans, vital signs measurements), that worsen from baseline, and is considered clinically significant in the medical and scientific judgment of the investigator (i.e., not related to progression of underlying disease).

- Exacerbation of a chronic or intermittent pre-existing condition, including either an increase in frequency and/or intensity of the condition.
- New conditions that are identified or diagnosed after the study treatment is administered, even though those conditions may have been present prior the start of the study.
- Signs, symptoms, or the clinical sequelae of a suspected drug-drug interaction.
- Signs, symptoms, or the clinical sequelae of a suspected overdose of a concomitant medication. Overdose per se will not be reported as an AE or SAE, unless it is an intentional overdose taken with possible suicidal/self-harming intent. Such overdoses should be reported regardless of sequelae.
- "Lack of efficacy" or "failure of expected physiological action" per se will not be reported as an AE or SAE. Such instances will be captured in the efficacy assessments. However, the signs, symptoms, and/or clinical sequelae, resulting from lack of efficacy, will be reported as AE or SAE, if they fulfil the definition of an AE or SAE.

6.3 Events Not Meeting the AE Definition

- Any clinically significant abnormal laboratory findings or other abnormal safety assessments that are associated with the underlying disease, unless determined by the investigator to be more severe than expected for the participant's condition.
- The disease/disorder being studied or expected progression, signs, or symptoms of the disease/disorder being studied, unless determined by the investigator to be more severe than expected for the participant's condition.
- Medical or surgical procedures (e.g., endoscopy, appendectomy): the condition that leads to the procedure is the AE.
- Events in which an untoward medical occurrence did not occur (social and/or convenience admission to a hospital).
- Anticipated day-to-day fluctuations of pre-existing disease(s) or condition(s) present or detected at the start of the study that do not worsen.

If an event is not an AE, as defined above, then it cannot be an SAE even if serious events occur (e.g., hospitalization for signs/symptoms of the disease under study; death resulting from progression of disease).

6.4 Events Meeting the Serious Adverse Event (SAE) Definition

- Results in death
- Is life threatening.
    - The term "life-threatening," in the definition of SAE, refers to the imminent risk of death of a participant at the time of the event. It does not refer to an event,

which hypothetically might have caused death of a participant, if it were more severe.

- Requires inpatient hospitalization or prolongation of existing hospitalization.
  - In general, "hospitalization" means the detainment of a participant at a hospital or emergency ward (usually involving at least an overnight stay) for observation and/or treatment, which would not have been appropriate in a physician's office or outpatient setting. Complications that occur during a participant's hospitalization are AEs. If such complications prolong the participant's hospitalization or fulfills any other SAE criteria, those complications are SAEs. When in doubt as to whether "hospitalization" occurred or was necessary, the AE should be considered serious. If a participant is hospitalized because of an elective treatment, of a preexisting condition that did not worsen from baseline, then the hospitalization is not considered an AE.
- Results in persistent disability/incapacity.
  - The term "disability" means a substantial disruption of a participant's ability to conduct normal life functions.
  - This definition does not include experiences of relatively minor medical symptoms (e.g., uncomplicated headache; nausea; vomiting; diarrhea; influenza; and accidental trauma [e.g., sprained ankle]) that may interfere with or even prevent daily life functions, but do not constitute a substantial disruption.
- Is a congenital anomaly/birth defect

The Investigator should exercise their medical and/or scientific judgment when deciding whether to report other situations that may cause participants to require medical or surgical treatment (but are not imminently life-threatening or resulting in death or hospitalization) as SAEs. These events should usually be considered serious.

Examples of such events include: invasive or malignant cancers; intensive treatment in an emergency room or at home for allergic bronchospasm; blood dyscrasias or convulsions that do not result in hospitalization; development of drug dependency; or drug abuse.

6.5 Adverse Event Severity

The investigator will assess the intensity of each AE or SAE that arises during the course of the study and assign each event one of the following categories:

- **Mild**: The event causes the participant minimal discomfort and does not interfere with the participant's normal daily activities;
- **Moderate**: The event causes the participant sufficient discomfort to interfere with the participant's normal daily activities;

- **Severe**: The event causes the participant from performing his or her normal daily activities. Investigators should note that a severe AE assessment should not be confused with an SAE assessment. "Severe" is a category utilized for rating the intensity of an event; both AEs and SAEs can be assessed as being "severe".

An event is defined as SAE when it meets at least one of the predefined outcomes as described in the definition of an SAE, NOT when it is rated as "severe."

6.6 Relationship to Study Intervention

The assessment of causality is one of the criteria used when determining regulatory reporting requirements. The Investigator must use their clinical judgement to assess whether there is a causal relationship between the study treatment and each AE or SAE.

- Probably Related: An AE that might be due to the study intervention. The relationship in time is suggestive. An alternative explanation is less likely, e.g., concomitant drug(s), concurrent disease(s).
- Possibly Related: An AE that might be due to the study intervention. The relationship in time is reasonable; therefore, a causal relationship cannot be excluded. An alternative explanation is inconclusive, e.g., concomitant drug(s), concurrent disease(s).
- Not Related: An AE is not thought to be due to the study intervention.

The Study Investigator should consider and investigate alternative causes (e.g., underlying disease(s); concomitant therapy; and other risk factors), as well as the temporal relationship of the event and study treatment administration, in their assessment of causality. For each AE or SAE, investigators must document, in the medical notes, that they have reviewed the AE or SAE and have provided an assessment of causality. Investigators may change their assessment of causality based on follow-up information.

6.7 Serious Adverse Event Reporting

All SAEs shall be reported to the Sponsor or designee within 24 hours of notification of the event. If the investigator receives any updates concerning SAEs, they shall submit such updates to the Sponsor within 24 hours of update receipt. Although the investigator is not required to actively seek out AEs or SAEs after participants have been discharged from the study, if the investigator learns of any study-related SAEs (including death), they must promptly notify the Sponsor of such SAEs.

SAEs will be reported to the Sponsor or designee via secure email. The investigators must also report SAEs to the IRB as required by their IRB regulations.

6.8 Following up on Adverse Events

The investigators must follow-up on each device-related SAE by performing or arranging for appropriate supplemental measurements and/or evaluations. This may include performing additional laboratory tests, histopathological examinations, or consulting with other health care professionals.

The CRC or other delegated team member shall record new or updated SAE-related information in a Case Report Form (CRF) hosted in the electronic source.

The investigators will be required to follow-up with each participant involved in AEs and SAEs at each subsequent visit or contact until a resolution or an explanation of the SAEs, or the participant stabilizes, is lost to follow-up, or for up to one week after the participant completes the study in the case of unresolved events.

6.9 Adverse Device Effects

An adverse device effect (ADE) is an adverse event related to the use of an investigational medical device. This includes any adverse event resulting from insufficiencies or inadequacies in the instructions for use, the deployment, the implantation, the installation, the operation, or any malfunction of the investigational medical device. This includes any event that is a result of a use error or intentional misuse and any event that has the potential for death or serious injury if the malfunction reoccurred.

Serious ADE definition: an ADE that has resulted in any of the consequences characteristic of a serious adverse event (SAE) that led to:

1) death
2) fetal distress, fetal death or a congenital abnormality or birth defect or
3) a serious deterioration in health that either resulted in
    - a life-threatening illness or injury, or
    - a permanent impairment of a body structure or a body function, or required in-patient hospitalization or prolongation of existing hospitalization, or
    - medical or surgical intervention to prevent life threatening illness or injury or permanent impairment to a body structure or a body function.

Documenting ADEs

The Study Investigator or other delegated team member shall identify, document, and report any ADE and malfunction resulting in an ADE that occurs any time during the study. After participants have been discharged from the study, if investigators learn of any study-related ADEs, they must promptly notify the Sponsor of such ADEs.

Follow-up of ADEs

Study Investigators or other delegated team member shall follow-up on all ADEs (including those involving participants who have been discharged from the study) involving an AE and report in accordance with the above sections. Investigators will ensure that supplemental investigations are conducted in all follow-ups to help determine the nature and/or causality of ADEs. Investigators shall update, sign, and date the ADE reports as new and additional follow-up information become available.

Adverse Device Effect Reporting

The sponsor assumes responsibility for appropriate reporting of ADEs to the regulatory authorities. The sponsor will also report suspected unanticipated SADEs to clinical site investigators. The investigator (or sponsor where required) must report unanticipated SADEs to the IRB that approved the protocol unless otherwise required and documented by the IRB.

All SADEs and SAEs occurring during the study must be reported to the appropriate sponsor contact person by study-site personnel within twenty-four (24) hours of their knowledge of the event.

7. Participant Compensation

All participants will be compensated for participation based on completion of study milestones depending on study activities completed.

8. Study Terminations

Participants are free to withdraw from the study at any time. The study principal investigator may exercise judgment to terminate a participant's involvement in the study if it is felt that continued participation might be harmful to the participant.

9. Sample Size

The primary study population consists of approximately 20 male and female adult participants (18 – 75 years old), with a diagnosis of nonalcoholic fatty liver disease (NAFLD) or nonalcoholic steatohepatitis (NASH), and who satisfy the inclusion and exclusion criteria. Only those who meet PDFF $\geq 10\%$ will be included in the primary endpoint analysis.

Sample size calculations are estimated for the primary analysis population, i.e. those who satisfy all inclusion/exclusion criteria and have a baseline PDFF $\geq 10\%$.

The sample size was calculated by referencing the mean absolute change in steatosis and standard deviation reported in a 2019 JAMA review of the impact to behavioral weight loss interventions on clinical markers of NAFLD (Koutoukidis et al., 2019). The average mean absolute change in steatosis was -2.4 and mean standard deviation was 1.6. Using these results, it was determined a sample size of approximately 8 participants would provide approximately 90% power, assuming an alpha of 0.05. To account for up to a 20% attrition rate, a minimum of 10 participants are required.

To ensure inclusion of a mix of patients with either NAFLD or NASH, a total of approximately 20 participants will be enrolled. It's expected that up to 50% of fully eligible participants will fail to meet the MRI-PDFF requirements for inclusion in the primary analysis population, resulting in approximately 10 participants included in the primary endpoint analysis. Participants who do not meet the MRI-PDFF requirement for primary analysis endpoint will be included in secondary and exploratory analyses.

Demographics and baseline characteristics will be summarized descriptively for all subjects based on the analysis population. Descriptive summaries will include the number of non-missing observations, mean, median, standard deviation, minimum and maximum for continuous results. Categorical results will be summarized with counts and percentages.

10. Analysis Populations

For purposes of analysis, the following populations are defined.

- Enrolled: All participants who complete the onboarding and first time user experience steps within the App for the first treatment cycle assigned. This population will be utilized for all safety summaries;
- Per Protocol: Participants who are enrolled and complete a minimum number of lessons in the App during the intervention period, as pre-specified in the Statistical Analysis Plan (SAP). This population of participants will be the Intent to Treat (ITT) group for analysis;

11. Statistical Analysis

SAS will be used to perform the statistical analyses. The general approach to analysis is described below. A full SAP, which documents and details all data handling rules and prospective statistical analyses, will be finalized prior to completion of participant enrollment.

Baseline and demographic characteristics will be summarized descriptively. The study's demographic characteristics, baseline values, the primary outcome and secondary outcome will

be summarized descriptively through the tabular display of mean values, medians, ranges, and standard deviations for continuous variables of interest and frequency distributions for categorical variables.

11.1 Primary and Secondary Endpoint Analyses

The primary efficacy endpoint is the change in percent liver fat as measured by MRI-PDFF from baseline to 3 months after enrollment. The results at baseline, at 3 months post-enrollment, and the change from baseline will be summarized descriptively. Change from baseline results will be calculated as (post-baseline minus baseline). The baseline result is defined as the last assessment prior to starting the Better Therapeutics App. In addition, the change from baseline in the percentage liver fat will be tested using a one-sample t-test if the normality assumption is met. The normality assumption will be assessed using the Shapiro-Wilks test for normality. If the normality assumption is not met, then the change from baseline will be tested using a Wilcoxon rank sum test.

The continuous secondary endpoints will be summarized in the same manner as described for the primary efficacy endpoint.

The categorical secondary endpoints, such as the percentage of participants who achieve ≥ 30% reduction in MRI-PDFF from baseline to post-intervention, will be summarized using counts and percentages. In addition, exact binomial confidence intervals for the true rates will be provided.

11.2 Exploratory Endpoint Analyses

The same statistical methods used to analyze the primary and secondary endpoints will be used to assess the exploratory endpoints. Mean changes in clinical values will be assessed from baseline to 12 weeks after enrollment.

11.3 Safety Summaries

Treatment-emergent Adverse Events will be summarized using counts and percentages for the participants in the Enrolled Population. A treatment-emergent adverse event (TEAE) will be defined as any AE that occurs after the start of use of the Better Therapeutics App. Summaries of AEs will include:

- All TEAEs;
- Serious TEAEs;
- TEAEs by type of event, severity and relationship to treatment
- and adverse device effects (ADE)

12. Data Storage

Data collected by the study site will be stored in CRIO, a secure and HIPAA-compliant electronic data management system.

Data generated through use of BT-NCBT-00x will be stored on the Better Therapeutics servers. The data storage is hosted by a third party, Amazon Web Services, which has a range of certifications, including HITRUST, SOC 2 Type II and ISO 27001, and using their HIPAA eligible services. The AWS platform is designed to protect subjects from threats by applying security controls at every layer from physical to application, isolating subject applications and data, with the ability to deploy security updates rapidly without subject interaction or service interruption. All subject data is transferred to AWS using industry standard Transport Layer Security (TLS). Subject data are password protected, encrypted at rest using Advanced Encryption Standard (AES) and only accessible by study personnel who have the proper authorization.

13. Confidentiality

This study will take extensive steps to protect confidentiality of participant information. First, upon completing the consent process, participants will be assigned a unique ID number which will be used when completing analysis and reporting results. This unique ID number will be the only identifier used in all data collection sources. Data containing these identification numbers will be stored separately from participants’ names and contact information. This measure is intended to ensure participant confidentiality. Data from the app servers will only be provided to authorized study personnel for the analysis of study outcomes and will be de-identified.

14. Risks and Benefits

The risks from participating in this study are minimal. The App, BT-NCBT-00x, asks questions about beliefs related to personal health that participants may find create some personal discomfort in answering. BT-NCBT-00x will provide recommendations for increased consumption of fruits, vegetables, whole grains, nuts, seeds, beans and legumes. The increased consumption of these types of foods could result in increased gastrointestinal gas and some physical discomfort.

BT-NCBT-00x also encourages gradual increases in physical activity of the participants' choosing. The engagement in physical activity comes with some risk for injury. However, the level of activity recommended by BT-NCBT-00x is consistent with standard of care guidelines and does not introduce risk beyond what is experienced in standard care.

If participant has comorbid conditions including type 2 diabetes or hypertension, and responds favorably to BT-NCBT-00x and make substantive changes while on medications for reducing blood glucose and/or blood pressure, the participant may experience transient and reversible drops in blood glucose and/or blood pressure that may require the participant to decrease medication usage under the guidance of a physician. Encouraging the participant to engage in self-monitoring of these biomarkers, if applicable, while using BT-0x is intended to mitigate these risks by enhancing the participant's awareness of blood glucose or blood pressure changes. Participants will be given the opportunity to self-monitor blood glucose and/or blood pressure if they indicate the presence of these conditions in treatment onboarding.

Engagement with BT-NCBT-00x may result in positive changes in behavioral habits related to diet, exercise and the management of stress. The results of changes in weight will be available to participants to view within BT-NCBT-00x and may provide participants insight into their health.

Delegated staff members at Arizona Liver Health will refer patients to the study and are registered study associates. If an AE is reported or discovered by a study associate, they will ensure participants receive appropriate care for any AEs experienced during the study period irrespective of their cause.
Given that the potential risks incurred in this study are minimal and there is a chance for increased knowledge and capacity for health promoting behaviors, the risk-to-benefit ratio is weighted towards greater benefits.

15. Ethical Guidelines and Institutional Review Board

The study will be conducted in alignment with the guidelines of the International Conference on Harmonisation for Good Clinical Practice (ICH-GCP). The study will be registered on http://ClinicalTrails.gov. De-identified summary results of this study may be submitted for publication in a peer-reviewed journal or presented at a professional conference.

Prior to initiating the study, Arizona Liver Health will obtain written confirmation from the institutional review board (IRB) that the study is properly constituted and compliant with Good Clinical Practice (GCP) requirements, applicable laws and local regulations. Arizona Liver Health will provide the IRB with all appropriate material, including, but not limited to, the protocol, Informed Consent Form (ICF) and any other information provided to the study participants.

16. References

Berman, M. A., Guthrie, N. L., Edwards, K. L., Appelbaum, K. J., Njike, V. Y., Eisenberg, D. M., & Katz, D. L. (2018). Change in Glycemic Control With Use of a Digital Therapeutic in Adults With Type 2 Diabetes: Cohort Study. *JMIR Diabetes*, *3*(1), e4. https://doi.org/10.2196/diabetes.9591

Cotter, T., & Rinella, M. (2020). Non-alcoholic fatty liver disease 2020: The State of the Disease. *Gastroenterology*, *158*(7), 1851–1864.

Guthrie, N. L., Appelbaum, K. J., & Berman, M. A. (2020). SAT-LB120 A Software Application Delivering Behavioral Therapy Improved Glycemic Control in Adults With Type 2 Diabetes. *Journal of the Endocrine Society*, *4*(Supplement_1), SAT-LB120. https://doi.org/10.1210/jendso/bvaa046.2152

Guthrie, N. L., Appelbaum, K. J., Simmons, M. L., Mehta, P., Rodbard, D., Katz, D. L., & Berman, M. A. (2020). *Impact of nutritional-CBT delivered by a mobile application in adults with type 2 diabetes*. 11.

Guthrie, N. L., Berman, M. A., Edwards, K. L., Appelbaum, K. J., Dey, S., Carpenter, J., Eisenberg, D. M., & Katz, D. L. (2019). Achieving Rapid Blood Pressure Control With Digital Therapeutics: Retrospective Cohort and Machine Learning Study. *JMIR Cardio*, *3*(1), e13030. https://doi.org/10.2196/13030

Hallsworth, K., & Adams, L. A. (2019). Lifestyle modification in NAFLD/NASH: Facts and figures. *JHEP Reports*, *1*(6), 468–479. https://doi.org/10.1016/j.jhepr.2019.10.008

Johnson, N. A., Sachinwalla, T., Walton, D. W., Smith, K., Armstrong, A., Thompson, M. W., & George, J. (2009). Aerobic exercise training reduces hepatic and visceral lipids in obese individuals without weight loss. *Hepatology (Baltimore, Md.)*, *50*(4), 1105–1112. https://doi.org/10.1002/hep.23129

Koutoukidis, D. A., Astbury, N. M., Tudor, K. E., Morris, E., Henry, J. A., Noreik, M., Jebb, S. A., &

Aveyard, P. (2019). Association of Weight Loss Interventions With Changes in Biomarkers of Nonalcoholic Fatty Liver Disease: A Systematic Review and Meta-analysis. *JAMA Internal Medicine*. https://doi.org/10.1001/jamainternmed.2019.2248

Polyzos, S. A., Kountouras, J., & Mantzoros, C. S. (2019). Obesity and nonalcoholic fatty liver disease: From pathophysiology to therapeutics. *Metabolism - Clinical and Experimental*, *92*, 82–97. https://doi.org/10.1016/j.metabol.2018.11.014

Younossi, Z., Anstee, Q. M., Marietti, M., Hardy, T., Henry, L., Eslam, M., George, J., & Bugianesi, E. (2018). Global burden of NAFLD and NASH: Trends, predictions, risk factors and prevention. *Nature Reviews Gastroenterology & Hepatology*, *15*(1), 11–20. https://doi.org/10.1038/nrgastro.2017.109

Younossi, Z. M., Corey, K. E., & Lim, J. K. (2021). AGA Clinical Practice Update on Lifestyle Modification Using Diet and Exercise to Achieve Weight Loss in the Management of Nonalcoholic Fatty Liver Disease: Expert Review. *Gastroenterology*, *160*(3), 912–918. https://doi.org/10.1053/j.gastro.2020.11.051

Younossi, Z. M., Golabi, P., de Avila, L., Paik, J. M., Srishord, M., Fukui, N., Qiu, Y., Burns, L., Afendy, A., & Nader, F. (2019). The global epidemiology of NAFLD and NASH in patients with type 2 diabetes: A systematic review and meta-analysis. *Journal of Hepatology*, *71*(4), 793–801. https://doi.org/10.1016/j.jhep.2019.06.021

17. APPENDICES

Appendix 17.1: Study Enrollment Landing

Better

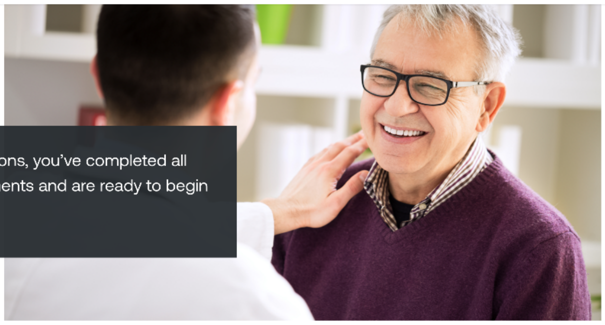

Congratulations, you've completed all the requirements and are ready to begin the study!

Better THERAPEUTICS   ARIZONA LIVER HEALTH

This study is exploring a behavioral treatment for nonalcoholic fatty liver disease (NAFLD) and nonalcoholic steatohepititis (NASH).

Start Now →

What does a study participant need to do?

- Download the mobile app (Android or iOS)
- Complete weekly lessons designed to help you improve your liver health and examine ideas that may be holding you back
- Learn skills and practice new ways of eating and living
- Track your health numbers and get feedback
- Return to Arizona Liver Health clinic in 3 months to repeat blood work and imaging

Have questions?
Contact the Study Coordinator at
(480) 470-4000

Create account →

How the treatment works

Exploring a different type of treatment
Instead of adding another pill, a behavioral treatment provided by an app helps you start to shift your beliefs and daily actions to develop a new way of living that may improve fatty liver disease.

Evidence-based approaches
Identify thoughts and beliefs getting in the way of improving your health. Cognitive exercises help you change your mindset and establish new routines.

Personalized feedback
As you track your actions and health numbers, you'll get feedback on your progress that will help you identify what's working for you and what may need more adjustment.

Start now →

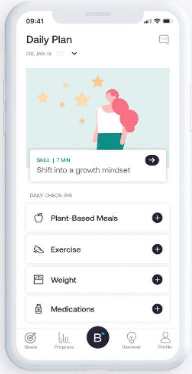

Better   research@bettertx.com   Terms   Privacy

Appendix 17.2: BT-NCBT-00x Digital Therapeutic (Daily Use)

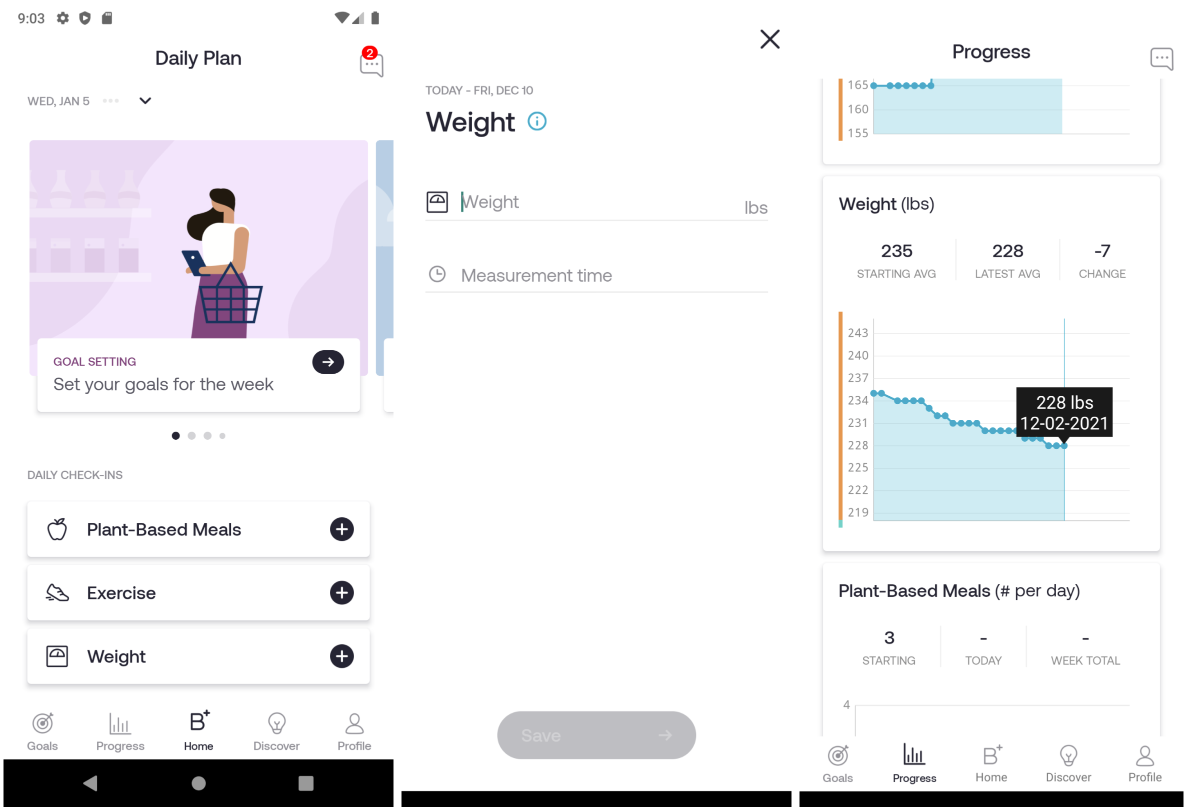

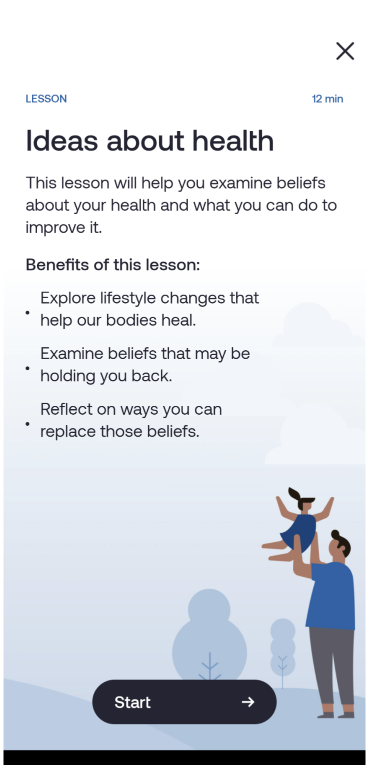
LESSON
12 min
Ideas about health
This lesson will help you examine beliefs about your health and what you can do to improve it.
Benefits of this lesson:
Explore lifestyle changes that help our bodies heal.
Examine beliefs that may be holding you back.
Reflect on ways you can replace those beliefs.
Start

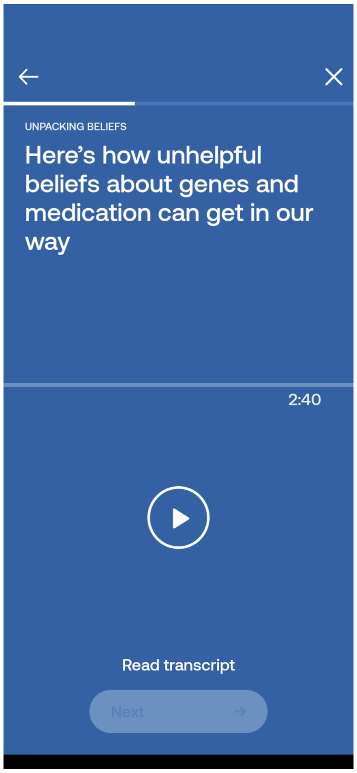
UNPACKING BELIEFS
Here's how unhelpful beliefs about genes and medication can get in our way
2:40
Read transcript
Next

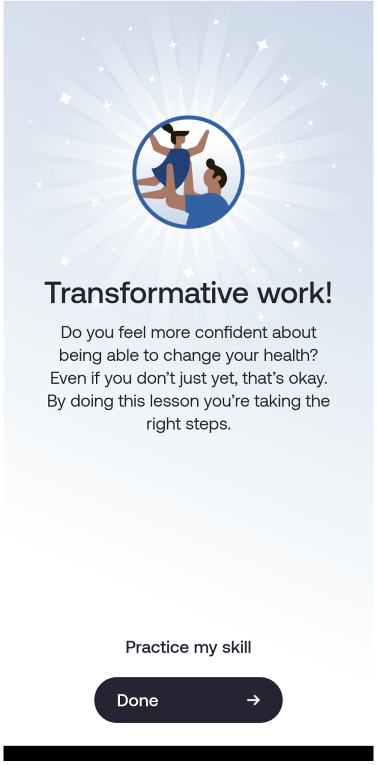
Transformative work!
Do you feel more confident about being able to change your health? Even if you don't just yet, that's okay. By doing this lesson you're taking the right steps.
Practice my skill
Done

Appendix 17.3: Behavioral Support Outreach and Scheduling

Support Message

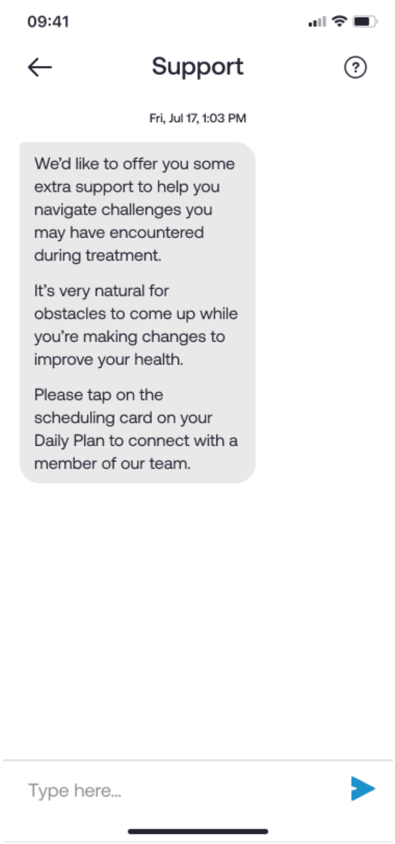

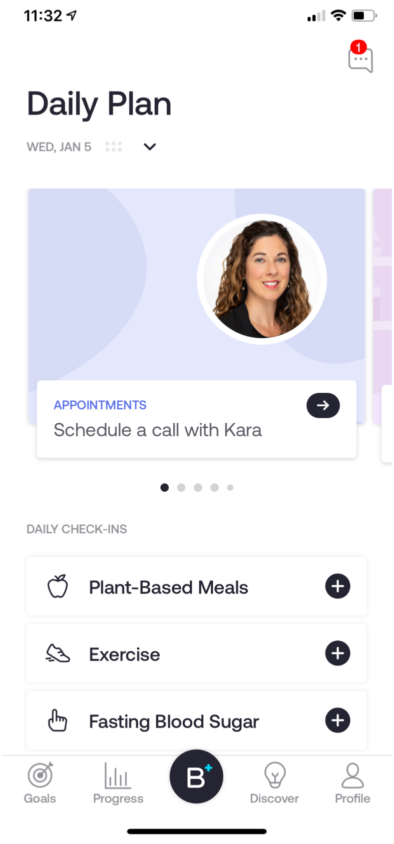

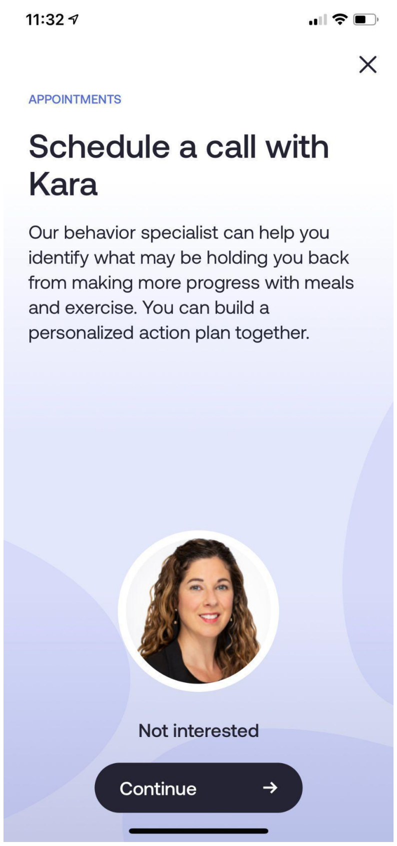

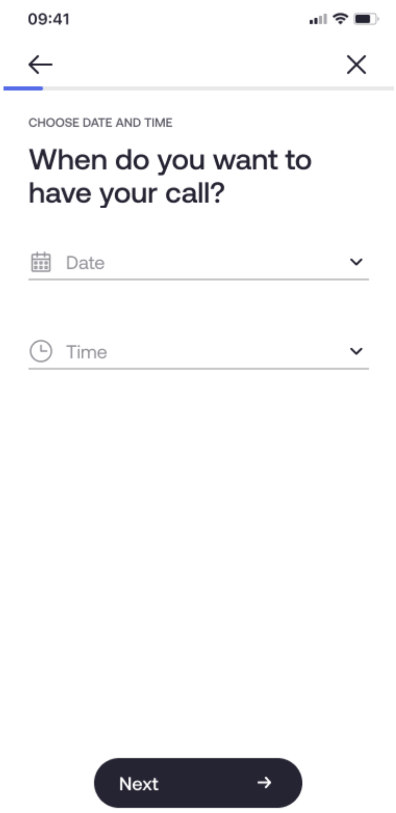
09:41
CHOOSE DATE AND TIME
When do you want to have your call?
Date
Time
Next

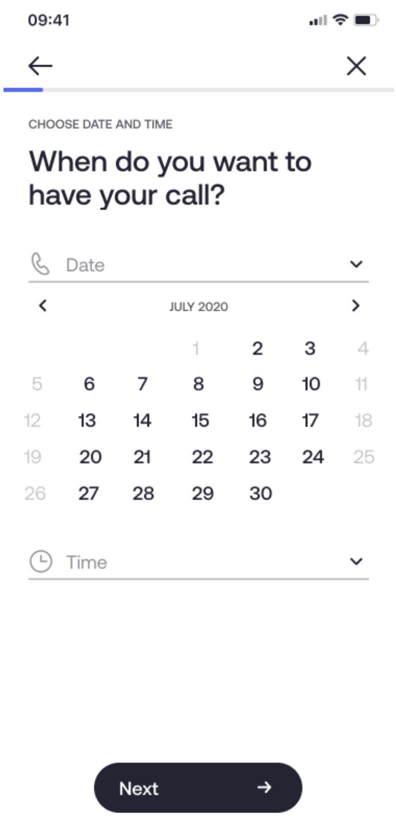
09:41
CHOOSE DATE AND TIME
When do you want to have your call?
Date
JULY 2020
1 2 3 4
5 6 7 8 9 10 11
12 13 14 15 16 17 18
19 20 21 22 23 24 25
26 27 28 29 30
Time
Next

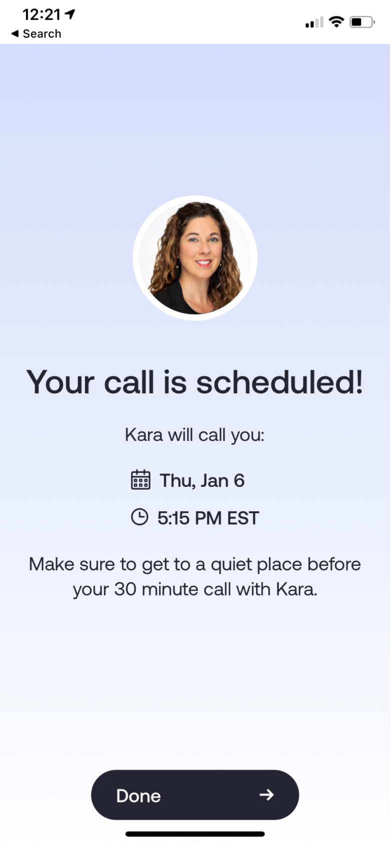
12:21
Search
Your call is scheduled!
Kara will call you:
Thu, Jan 6
5:15 PM EST
Make sure to get to a quiet place before your 30 minute call with Kara.
Done

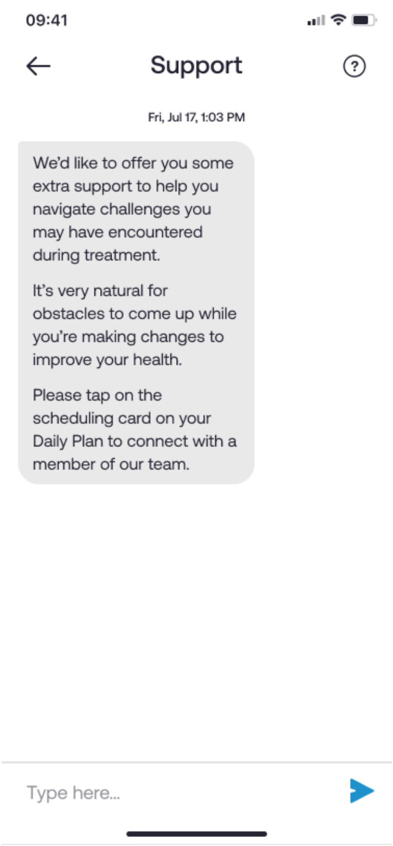
09:41
Support
Fri, Jul 17, 1:03 PM
We'd like to offer you some extra support to help you navigate challenges you may have encountered during treatment.
It's very natural for obstacles to come up while you're making changes to improve your health.
Please tap on the scheduling card on your Daily Plan to connect with a member of our team.
Type here...

Appendix 17.4: BT-NCBT-00x Agnostic Treatment Experience Survey

[Bracketed text is internal labeling and is not shown to participants]

Your treatment experience

[INTRO]

Now that you're getting close to finishing this 90 day cycle of treatment, it would be very helpful to hear your feedback about your experience.

Please do your best to answer all the questions, we really appreciate it.

[Q1, select step]
If given the option, would you like to continue using this treatment for another 90 day cycle?

- ☐No
- ☐Yes

[Q2, select step]
What reasons would influence your decision to continue or discontinue the use of the treatment? You can select more than one.

- ☐I don't need this treatment because I met my health goals
- ☐I'm still working on meeting my health goals (example: blood sugar, weight)
- ☐I need this treatment to maintain the improvements I have made
- ☐My healthcare provider thinks I should keep using it
- ☐The treatment was not helpful
- ☐Other ______[free text]___________

[Q3, select step]
Which parts of this treatment **helped you the most**? You can select more than one.

- ☐Articles
- ☐Daily Check-Ins for blood sugar, blood pressure or weight
- ☐Daily Check-Ins for meals and exercise
- ☐Goal Setting
- ☐Lessons
- ☐Progress Reports
- ☐Recipes
- ☐Skills
- ☐None of the above
- ☐Other ______[free text]________________

[Q4, select step]
Which parts of this treatment were **not helpful to you**? You can select more than one.

- ☐ Articles
- ☐ Daily Check-Ins for blood sugar, blood pressure or weight
- ☐ Daily Check-Ins for meals and exercise
- ☐ Goal Setting
- ☐ Lessons
- ☐ Progress Reports
- ☐ Recipes
- ☐ Skills
- ☐ None of the above
- ☐ Other______[free text]________________

[Q5, select step]
Have you shared any health data from this treatment with your primary care provider?

- ☐ No
- ☐ Yes

[Q5b, write step - skippable]
What kind of data was most useful to share?

[Q6, write step - skippable]
What was your biggest takeaway from the last 90 days of using this treatment?

[Q7, select step]
Would you be interested in participating in a call with our product team to offer feedback on your experience using the treatment? You would be compensated for your time.

- ☐ No
- ☐ Yes

[Q8, select step]
Would you like our team to contact you regarding other research opportunities in the future?

- ☐ No
- ☐ Yes

[Final Screen]
Thank you for sharing!
Your feedback helps us improve this treatment for everyone.

Q1. Would you say that in general your health is

- Excellent [1]
- Very Good [2]
- Good [3]
- Fair [4]
- Poor [5]
- Don’t know/Not Sure [7]

Q2. Now thinking about your physical health, which includes physical illness and injury, for how many days during the past 30 days was your physical health not good?

- Number of Days ___________ [numerical entry, min 1, max 30]
- None [8 8]
- Don’t know/Not Sure [7 7]

Q3. Now thinking about your mental health, which includes stress, depression, and problems with emotions, for how many days during the past 30 days was your mental health not good?

- Number of Days ___________ [numerical entry, min 1, max 30]
- None [8 8]
- Don’t know/Not Sure [7 7]

Q4. During the past 30 days, for about how many days did poor physical or mental health keep you from doing your usual activities, such as self-care, work, or recreation?

- Number of Days ___________ [numerical entry, min 1, max 30]
- None [8 8]
- Don’t know/Not Sure [7 7]

Appendix 17.6: Net Promoter Score (NPS)

A standardized tool for measuring an individual's satisfaction with the program. It is asked at Day 70 in the behavioral app.

<u>The Question</u>

How likely are you to recommend [*NAME OF APP]* to a friend with a relevant health condition?

Enter a number 1 to 10 (1 being "No Way" to 10 being "Absolutely")

<u>The Scoring</u>

1. Assign each score a category
    - 0 to 6 = Detractor
    - 7, 8 = Passive
    - 9, 10 = Promoter

2. Sum the frequency of each category and calculate the % of total responses

3. NPS = % Promoters - % Detractors
