# Supplementary material for: A Novel Prescription Digital Therapeutic Option for the Treatment of Metabolic Dysfunction-Associated Steatotic Liver Disease
Source: Gastro Hep Adv. 2023 Oct 1;3(1):9–16. doi: 10.1016/j.gastha.2023.08.019 (PMC11307699; doi:10.1016/j.gastha.2023.08.019)
Supplement: Table A1 [file mmc2.docx]

Supplementary Materials

**Supplementary Table 1: Adverse events reported in the safety population, n=22**

|  | Subjects  n (%) | Events  n | Mild | Moderate | Severe |
| --- | --- | --- | --- | --- | --- |
| An Adverse Event (AE) | 6 (27%) | 10 | 5 | 5 | 0 |
| Relatedness  Possibly/Probably  Related to Study  Intervention  Is Related to Medical  Device | 0 (0%)  0 (0%) | 0  0 | -  - | -  - | -  - |
| Serious Adverse Event (SAE) | 0 (0%) | 0 | - | - | - |
