# Supplementary figures and images for: A Novel Prescription Digital Therapeutic Option for the Treatment of Metabolic Dysfunction-Associated Steatotic Liver Disease
Source: Gastro Hep Adv. 2023 Oct 1;3(1):9–16. doi: 10.1016/j.gastha.2023.08.019 (PMC11307699; doi:10.1016/j.gastha.2023.08.019)

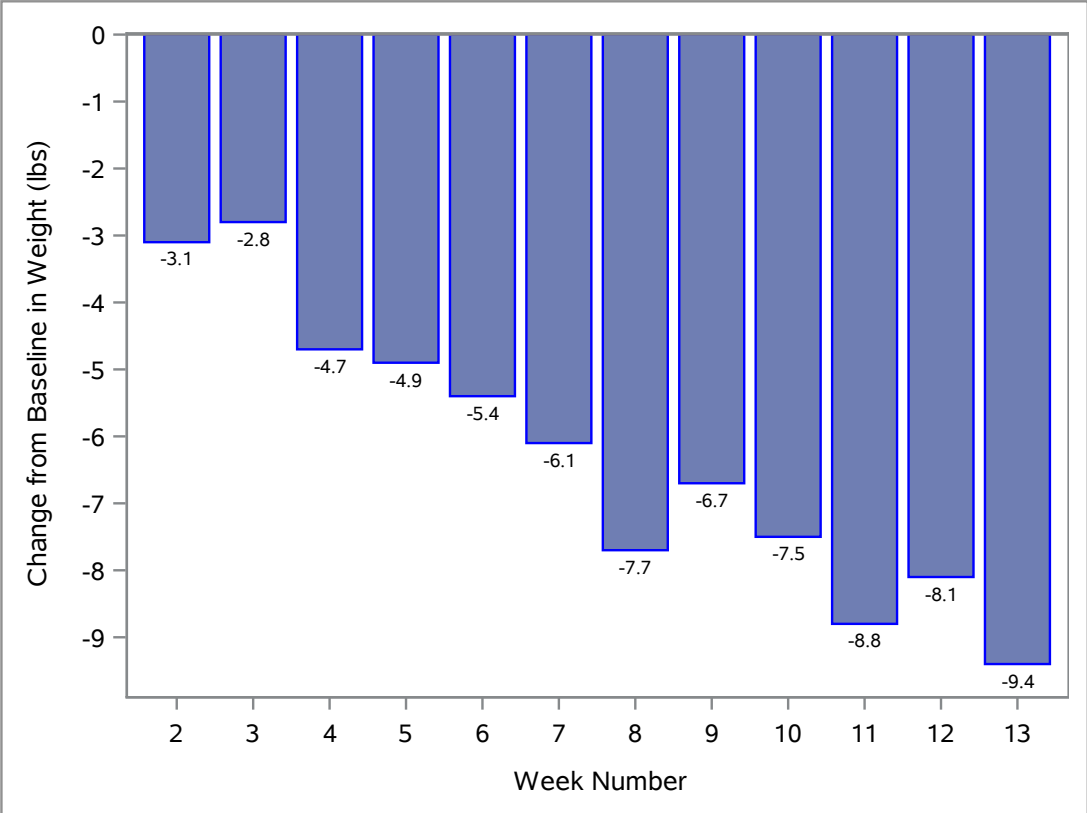

Supplement: Figure A1 [file mmc3.pdf]
