# Supplementary material for: A Novel Prescription Digital Therapeutic Option for the Treatment of Metabolic Dysfunction-Associated Steatotic Liver Disease
Source: Gastro Hep Adv. 2023 Oct 1;3(1):9–16. doi: 10.1016/j.gastha.2023.08.019 (PMC11307699; doi:10.1016/j.gastha.2023.08.019)
Supplement: Legend of Figure A1 [file mmc4.docx]

Supplementary Figure Legend

**Supplementary Fig. 1: Mean Self-Reported Weekly Weight Change**
An average of 5.0 (n=17, SD=2.5) self-reported weight values were logged per patient per week in the PDT over the
13-week intervention. The pattern of weight loss observed indicates a steady and consistent pattern of weight loss
with no apparent peak or plateau.
